# Role of Endophytic Entomopathogenic Fungi in Mediating Host Selection, Biology, Behavior, and Management of Tarnished Plant Bug, *Lygus lineolaris* (Hemiptera: Miridae)

**DOI:** 10.3390/plants13152012

**Published:** 2024-07-23

**Authors:** Justin George, James P. Glover, Omaththage P. Perera, Gadi V. P. Reddy

**Affiliations:** Southern Insect Management Research Unit, Agricultural Research Service, United States Department of Agriculture, Stoneville, MS 38776, USA; james.glover@usda.gov (J.P.G.); op.perera@usda.gov (O.P.P.); gadi.reddy@usda.gov (G.V.P.R.)

**Keywords:** fungal endophytes, entomopathogens, *Beauveria bassiana*, plant volatiles, host plant selection, cotton, host plant resistance

## Abstract

Non-insecticidal control strategies using entomopathogens, nematodes, and endophytes provide sustainable and safer alternatives for managing crop pests. This study investigated the potential of different fungal endophytes, specifically *Beauveria bassiana* strains, in colonizing cotton plants and their efficacy against tarnished plant bug, *Lygus lineolaris*. The effect of endophytes on plant growth parameters and cotton yield were measured during different plant growth stages. The entomopathogenicity of these fungi was studied in diet cup bioassays using *L. lineolaris* adults. The behavior of adult males and females toward endophytic cotton squares was analyzed using olfactometer assays. The experiments showed that the fungal endophytes colonized the plant structures of cotton plants, which resulted in an increase in the number of cotton squares, plant height, and weight compared to control plants. *B. bassiana* strains/isolates such as GHA, NI-8, and JG-1 caused significant mortality in *Lygus* adults compared to controls. Also, male and female *Lygus* adults exhibited repellence behavior towards endophytic cotton squares containing JG-1 isolate of *B. bassiana* and to other *B. bassiana* strains such as NI-8, GHA, and SPE-120. No differences were observed in the survival and development of *L. lineolaris* second-instar nymphs on endophytic cotton, and no yield differences were observed in the field experiments.

## 1. Introduction

Endophytes are plant-associated microorganisms that colonize tissues and reproductive structures of plants for part of their life cycle without causing damage to their host [1,2]. These naturally occurring microorganisms are described as bacteria, fungi, archea, and protists. Among these groups, fungal and bacterial endophytes are the most studied taxa [3]. Fungal endophytes colonize above-ground plant tissues such as stems, leaves, flowers, and seeds [1,4], whereas mycorrhizal fungi colonize the plant rhizosphere. These fungal endophytes can also act as entomopathogens and play an essential role as biocontrol agents against a myriad of insect pests and pose little or no harm to non-target insects and other beneficial organisms [5,6]. Studies have reported that these fungal endophytes can improve the plant response to biotic and abiotic stress through induction of systemic resistance or the production of insecticidal, antifungal, or antiviral compounds, in addition to its effectiveness against insect and mite pests [7]. Fungal endophytes have also attracted the attention of the scientific community in recent years due to their potential beneficial effects on vegetation, including in the facilitation of plant growth in metal-polluted environments. Studies have reported that both fungal and bacterial endophytes can co-exist in the same leaf or stem tissues of the host plant [8,9]. Fungal endophytes have been reported in a variety of field crops, including vegetables, fruits, maize, cotton, coffee, jute, and cocoa [6].

Endophytes provide numerous benefits to their host plant. The application of facultative endophytes in promoting plant growth and pest management can be improved by artificial inoculation of endophytes into plants by seed treatment, surface spray, and root inoculation. These fungal endophytes may act as multifaceted tools in plant growth and protection from pests and diseases and facilitate improved nutrient transfer and increased yield [10,11,12]. Endophytes act as yield promoters, soil nutrient distributers, abiotic stress, and drought tolerance enhancers in plants, as well as an indirect defense for insect pests, nematodes, and diseases [6,13]. Some are known to serve as pathogenic agents in plants by infecting lepidopterous larvae, aphids, thrips, and other insect pests, which are of great concern in agriculture [14]. Endophytes can also act as plant-defending mutualists and provide protection against herbivores [15,16]. These endophytic entomopathogenic fungi, such as *B. bassiana*, *Clonostachys rosea*, *Metarhizium anisopliae*, and *Lecanicillium lecanii,* have been reported to control pests and reduce their damage in crops [17]. Colonization of corn and sorghum by *B. bassiana* resulted in reduced tunneling by *Ostrinia nubilalis* Hubner and *Sesamia calamistis* Hampson [18,19]. Studies have shown that cotton aphid, *Aphis gossypii* (Glover), populations are significantly reduced in cotton colonized by endophytic strains, *B. bassiana* Vuillemin and *Phialemonium inflatum* (Burnside) strain TAMU-490 [20,21]. Evidence has also shown that the development of the cotton bollworm *Helicoverpa zea* (Boddie) is significantly reduced when exposed to *B. bassiana* [21,22]. Because of the benefits endophytes could offer to agricultural crops, it is important to explore endophytes as an alternative control strategy in disease and pest management programs [6].

The effect of fungal endophytes on the performance and feeding behavior of insect herbivores, including phloem feeders such as aphids and whiteflies [20,23], dipterans [24], beetles [25,26], caterpillars [21,27,28,29], grasshoppers [30], and hymenopteran gall wasps [31], has been reported. Fewer studies have explored the behavioral responses of hemipteran pests towards fungal endophytic plants, as they directly feed on fruiting structures and cause economic damage to the crop. Polyphagous pests such as plant bugs (Miridae) and stink bugs (Pentatomidae) are a major problem in row crops, and their feeding results in significant yield loss. Sword et al. [32] reported that *Lygus hesperus* and *Nezara viridula* showed strong negative responses to flower buds and cotton bolls and were deterred prior to contact with endophyte-colonized plants.

The tarnished plant bug, *Lygus lineolaris* (Palisot de Beauvois) (Hemiptera: Miridae), is considered the most important economic pest of cotton in the mid-southern United States, feeding on more than 700 plant species [33]. It is the most widely established *Lygus* species found on agricultural crops in North America and was reported to infest 6.9 million acres of cotton in 2021. In this study, we investigated the endophytic and entomopathogenic activity of five different fungal endophytes and an arbuscular mycorrhizal fungus on the olfaction behavior, mortality, host plant resistance, and the pest biology of tarnished plant bug, *L. lineolaris*, a major cotton pest in the Mississippi delta. Greenhouse and field experiments explored the effect of these fungal endophytes on cotton growth-promoting factors, plant bug damage, and cotton yield.

## 2. Results

### 2.1. Plant Growth Measurements of Fungal Endophytic Plants

**Figure 1:**
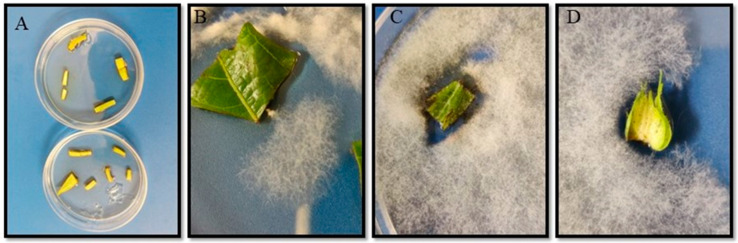
(**A**) Plant sample cultures collected from endophytic plants to test the presence of fungal endophytes in roots, stems, leaves, and squares of cotton plants; (**B**,**C**) fungal endophyte growing from leaves of endophytic plants; (**D**) fungus growing from squares of endophytic cotton plants.

Cotton plant growth parameters such as percentage germination, number of true leaves, cotton squares, plant height and dry root mass, stem and whole plant were measured to understand the effect of fungal endophytes on plant growth and physiology. No significant effects were observed in the percentage germination of different endophyte-coated seeds (*F*_6,277_ = 1.44, *p* = 0.19, *n* = 40) (Table 1). No differences were observed in the number of true leaves produced 14 days after germination (*F*_6,212_ = 1.21, *p* = 0.31, *n* = 30). The mean number of true leaves was in the range of 3.2–3.5 leaves/plant (Table 1). A significant difference was observed in the number of squares produced on the plants. The plants containing fungal endophyte SPE-120 produced a higher number of squares (4.2 ± 0.20) than the control plants (2.4 ± 0.24) and those containing other fungal endophytes studied (*F*_6,224_ = 6.64, *p* < 0.001, *n* = 32) (Table 1). TAMU-490 plants were the shortest in length compared to all the other plants studied (*F*_6,224_ = 11.92, *p* < 0.001, *n* = 32). No significant differences were observed in the dry weight of plant roots from the different endophytic plants (*F*_6,69_ = 1.66, *p* = 0.14, *n* = 10), whereas a significant effect was observed in the dry weight of stem and leaves (*F*_6,69_ = 12.10, *p* < 0.001, *n* = 10) (Table 1). The arbuscular mycorrhizal fungal (AMF) plants had the highest stem dry weight (30.5 ± 1.98) compared to control plants (17.3 ± 1.11). A similar trend was observed for the whole plant dry weight, and the AMF plants had the highest dry weight compared to control plants and other endophytic colonized plants (*F*_6,69_ = 11.12, *p* < 0.001, *n* = 10) (Table 1). Sporulation was observed on the various plant parts cultured such as leaf, stem, root, and squares (Figure 1), and the fungal spores were verified using Q-PCR.

### 2.2. Lygus lineolaris Mortality Following Direct Exposure to Fungal Spores

**Figure 2:**
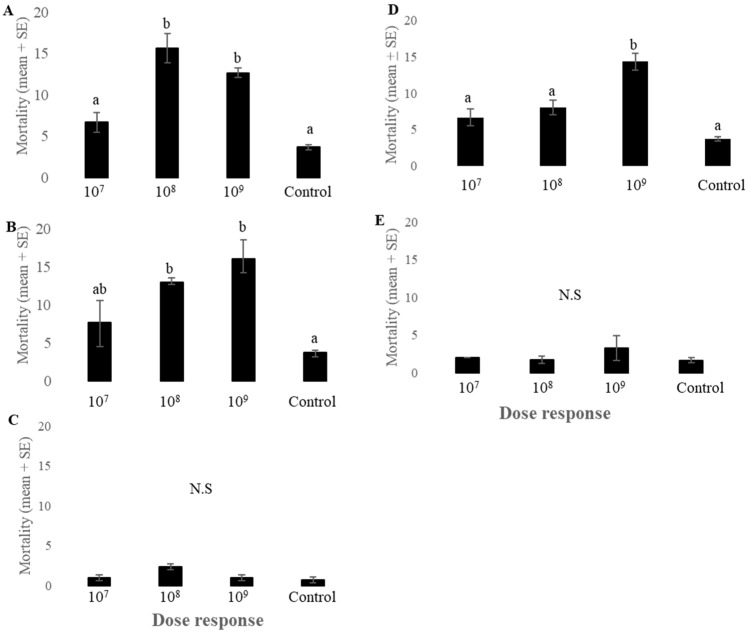
Mortality of *Lygus lineolaris* adults (mean ± SE) following exposure to different concentrations of endophytic fungal spores. (**A**) GHA, (**B**) JG-1, (**C**) TAMU-490, (**D**) NI-8 and (**E**) SPE-120 compared to water controls. Data were analyzed by ANOVA followed by Tukey’s HSD for mean comparisons (*n* = 30). Treatments that have no letters in common were significantly different (α = 0.05).

*Lygus lineolaris* adults exposed to different concentrations of endophytic fungal spores exhibited differential mortality rates. The commercial GHA strain of *B. bassiana* (BotaniGard 22 WP) showed significantly higher mortality of *L. lineolaris* adults at concentrations of 10^8^ and 10^9^ than the control (*F*_3,11_ = 25.12, *p* < 0.001, *n* = 3) (Figure 2A). No significant mortality effects of GHA strain of *B. bassiana* were observed at 10^7^ concentrations. The fungal endophyte JG-1 containing *B. bassiana* had a significant impact on the mortality of *L. lineolaris* adults at a concentration of 10^8^ and 10^9^ compared to controls (*F*_3,11_ = 7.93, *p* < 0.01, *n* = 3) (Figure 2B). Different concentrations of TAMU-490 and SPE-120 did not affect the mortality of *L. lineolaris* adults (*F*_3,11_ = 1.16, *p* = 0.38, *n* = 3) (*F*_3,11_ = 0.95, *p* = 0.45, *n* = 3, respectively) (Figure 2C,E). NI-8 showed significantly higher mortality of *L. lineolaris* at 10^9^ concentrations compared to the control and other concentrations tested (*F*_3,11_ = 20.18, *p* < 0.001, *n* = 3, respectively) (Figure 2D). Sporulation of *L. lineolaris* adult cadavers was observed for many of the fungal endophytes used in the study.

### 2.3. Detection and Validation of Endophytic Fungal Species in Treated Plants

Alignment of ITS1/2 nucleotide sequences from PDA cultures and stock fungal cultures indicated the presence of the fungal strains used for inoculation in stems and roots of cotton plants (Supplemental Figure S1a,b). ITS nucleotide sequences of different *B. bassiana* isolates contained nucleotide polymorphisms, insertions, or deletions specific to each isolate. More than 90% of the sequence reads generated from stock fungal isolates of *B. bassiana* (GHA, JG1, NI8, and SPE) and *P. inflatum* (TAMU) assembled to produce an ITS sequence specific to each fungal isolate. Nucleotide sequence reads generated from fungal cultures from cotton square tissues had 65–80% sequence reads mapping to the ITS sequences of corresponding stock isolates, indicating that the fungal inoculums had successfully established in the cotton plants. Nucleotide sequences of fungal cultures from square tissue that did not map to the stock isolates may indicate that either fungal endophytes other than the isolates used to inoculate seeds were also present in the square tissues or sequence reads had mismatches or low-quality nucleotide calls that did not meet the 96% similarity cut off. Evolutionary relationships among the ITS sequences of the fungal strains are shown in the Supplemental Figure S2. The branch representing *P. inflatum* was supported in 100% of the bootstrap replicates, while the bootstrap support for grouping different isolates of *B. bassiana* ranged from 0 (NI8) to 86 (SPE). This low support is due to the low number of differences between ITS nucleotide sequences of *B. bassiana* isolates.

### 2.4. Survival and Development of Lygus lineolaris Nymphs on Fungal Endophytic Plants

**Figure 3:**
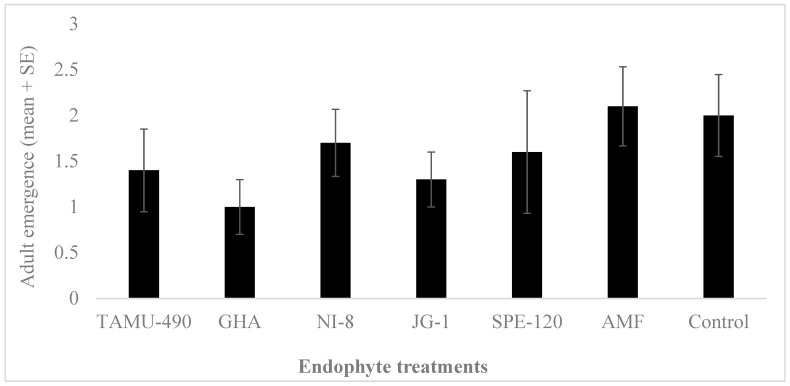
*Lygus lineolaris* adult emergence on endophyte-treated cotton plants (mean ± SE). Second instars were introduced to the plants, and adult emergence was recorded after 8 days. Data were analyzed by ANOVA using JMP software (v. 10, SAS Inc., Cary, NC, USA). No significant differences were observed in the adult emergence of *Lygus lineolaris* on different endophytic cotton plants.

The development of *L. lineolaris* nymphs on cotton pinhead squares was overall low for all the endophytic plants studied, including the control. No significant differences were observed in the development and adult emergence of *L. lineolaris* on these endophytic plants (*F*_6,69_ = 0.83, *p* = 0.55, *n* = 10) (Figure 3). A higher mortality of nymphs was observed on all the plants used in the experiments.

### 2.5. Olfactometer Studies Using Lygus lineolaris Adults Towards Endophytic Cotton Squares

**Figure 4:**
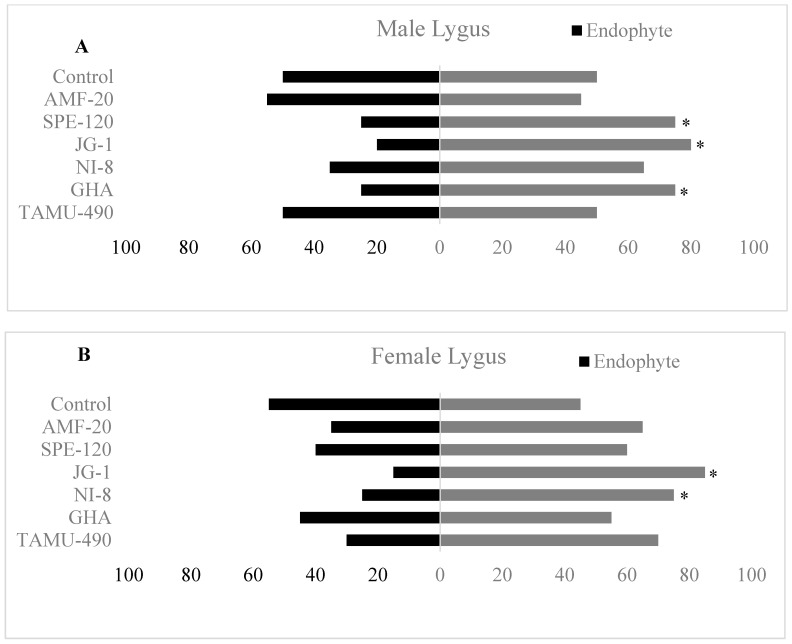
Behavioral response of *Lygus lineolaris* (**A**) males and (**B**) females towards endophytic cotton squares in olfactometer assays. * indicates a significant difference in their behavioral choice towards endophytic squares compared with control squares (α = 0.05).

Y-tube olfactometer studies investigated the behavioral responses of *L. lineolaris* male and female adults towards endophytic cotton squares. Male *L. lineolaris* adults showed significant repellence behavior towards cotton squares from fungal endophytes such as SPE-120, JG-1, and GHA compared to their corresponding controls ((χ^2^ = 5.0; df = 1; *p* = 0.025), (χ^2^ = 7.2; df = 1; *p* = 0.007), (χ^2^ = 5.0; df = 1; *p* = 0.025), respectively) (Figure 4A). No significant differences were observed in the behavior of male *L. lineolaris* towards cotton squares of TAMU-490, NI-8 and AMF compared to their controls. No differences were observed in the behavior of male *L. lineolaris* towards double controls that were performed to avoid any bias in their movement towards light or position preferences (Figure 4A).

Female *L. lineolaris* adults showed a significantly higher repellence behavior towards cotton squares from fungal endophytes such as JG-1 and NI-8 compared to their corresponding controls ((χ^2^ = 9.8; df = 1; *p* = 0.001), (χ^2^ = 5.0; df = 1; *p* = 0.025), respectively) (Figure 4B). No significant differences were observed in the behavior of female *L. lineolaris* adults towards cotton squares of AMF, SPE-120, TAMU-490 and GHA compared to their controls. No differences were observed in the behavior of female *L. lineolaris* towards double controls that were performed to avoid any bias in their movement towards light or position preferences (Figure 4B).

### 2.6. Performance of Fungal Endophytes against Tarnished Plant Bugs and Bollworms under Field Conditions

**Figure 5:**
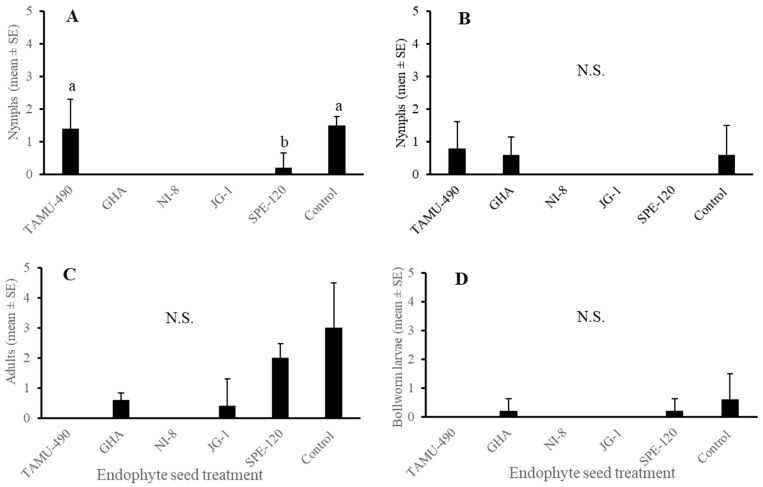
Mean (± SEM) *Lygus lineolaris* and *Helicoverpa zea* larval counts 3 d post-treatment. (**A**) first–third-instar *Lygus lineolaris*; (**B**) fourth- and fifth-instar *Lygus lineolaris*; (**C**) Adult tarnished plant bug; and (**D**) pooled *Helicoverpa zea* larvae. Tarnished plant bugs were recorded as small or large based on the absence or presence of wing pads. Bars with different letters indicate significant differences (α = 0.05) between treatments by model contrast analysis based on a generalized linear mixed-effect model.

Field sampling of tarnished plant bug nymphs and adults on plant terminals showed a significantly fewer number of first–third-instar nymphs on SPE-120-treated plants compared to TAMU-490 and control plants. No early instar nymphs were observed on GHA, NI-8, and JG-1 endophyte-treated plants (*p* < 0.05) (Figure 5A). No significant differences were observed in the number of late instar nymphs (4th–5th) or adults observed on different endophyte-treated plants and control (*p* > 0.05) (Figure 5B,C). Also, no differences were observed in the number of different instar cotton bollworm larvae observed on the treated and control plants (Figure 5D). Some treatment plants did not receive any stages of tarnished plant bugs or cotton bollworm larvae.

**Figure 6:**
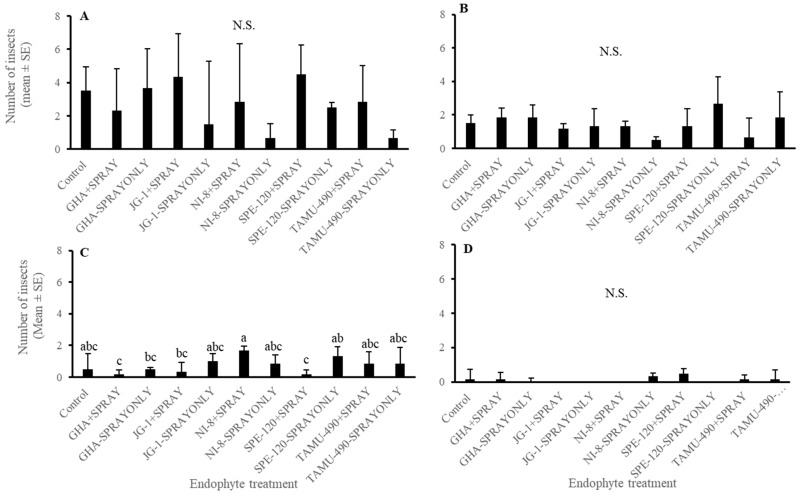
Mean (±SEM) number of second-instar *Lygus lineolaris* caged on the top five nodes of cotton plants in the second week of bloom and allowed to develop for one week. (**A**) Third-instar *Lygus lineolaris* nymphs; (**B**) Fourth-instar *Lygus lineolaris* nymphs; (**C**) Fifth-instar *Lygus lineolaris* nymphs; and (**D**) adult *Lygus lineolaris*. Bars with different letters indicate significant differences (α = 0.05) between treatments by model contrast analysis based on a generalized linear mixed-effect model.

**Figure 7:**
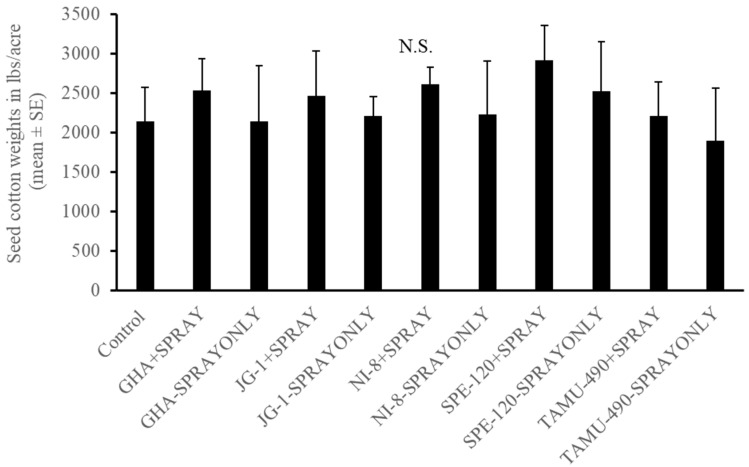
Mean (±SEM) seed cotton weights for seed coated and topical application of cotton endophyte-treated plants. Control plants were sprayed with water. Treatments consisted of seeds treated with spores of endophyte alone and in combination with a foliar endophyte spray. No significant differences (α = 0.05) observed between treatments by model contrast analysis based on a generalized linear mixed-effect model.

In the experiment where second-instar *L. lineolaris* nymphs were introduced in the top five nodes of cotton plants under field conditions, no differences were observed in the development of third-instar or fourth-instar plant bug nymphs (*p* > 0.05) (Figure 6A,B). A combination of seed treatment alone, foliar spray alone, or in combination had no effect on their development. Endophytic seed treatment of GHA combined with endophyte foliar spray and SPE-120 seed treatment combined with foliar spray had the lowest number of fifth-instar *L. lineolaris* nymphs than other treatments post threshold and foliar endophyte spray (*p* < 0.05) (Figure 6C). No differences were observed in the development and adult emergence of *L. lineolaris* on the different endophytic treatments (*p* > 0.05) (Figure 6D). Cotton was harvested from individual treatment plots. No significant differences were observed in the yield of cotton produced under different endophyte seed treated with spray alone or combination treatments (*p* > 0.05) (Figure 7). Even though the yield was numerically higher in SPE-120 seed + spray treatments than controls, the yield did not show a statistical difference.

## 3. Discussion

Ascomycetous insect-pathogenic fungi such as *Beauveria* and *Metarhizium* can act as endophytes and symbionts in addition to their pathogenicity against many arthropod species. These endophytic entomopathogenic fungi can influence plant growth and the infestation rate of different pests and diseases. Also, the endophytes may help in mitigating biotic and abiotic stress associated with plant growth and herbivore infestation. In this study, we tested the endophytic activity of different known and unknown strains of *B. bassiana* to investigate their effect on cotton growth characteristics, yield, pest infestation rates, behavioral responses of *L. lineolaris* adults to endophytic cotton squares, and yield parameters of different endophytic seed treatments and spray applications. Our PCR analysis showed that the different commercial strains (NI-8, BotaniGard 22 WP, SPE-120) and isolated strains (JG-1) of *B. bassiana* were able to successfully colonize as fungal endophytes on different plant tissues such as leaf, stem, roots, and fruiting squares of cotton plant (Figure 1 and Figure S1a,b). Prior studies have reported the epiphytic and endophytic growth of *B. bassiana* strain GHA on tomato plants [34].

Previous studies have shown that fungal endophytes such as *B. bassiana* and *P. inflatum* affect the host plant selection behavior of *Lygus hesperus* (western tarnished plant bug) and *Nezara viridula* (Southern stink bug), which are two major pests in cotton and soybean [32]. Our research investigated how the fungal endophytes and arbuscular mycorrhizal fungus affect plant growth and yield, as well as the development of *L. lineolaris* nymphs under controlled greenhouse and field conditions. We observed a significant difference in the number of cotton squares produced by *B. bassiana* endophyte strains such as SPE-120 and NI-8 compared to the controls. Simultaneously, no differences were observed in the number of squares by TAMU-490 and GHA *B. bassiana* strain compared to the controls. The stem dry weight and whole plant weight were significantly higher for arbuscular mycorrhizal fungus AMF than the control, TAMU-490, and some of the *B. bassiana* endophytes such as SPE-120, JG-1, and GHA. Previous studies have also reported that the hyphal network formation of AMFs contributes towards plant growth [35] and increased availability and translocation of nutrients [36]. No significant differences were observed in the root weight of AMF treatments compared to controls or other endophytes studied. It was observed that the AMFs had a lot more fibrous fine roots compared to other treatments.

Mortality assays using different concentrations of commercial, isolated *B. bassiana* strains, and *P. inflatum* showed high mortality of *L. lineolaris* adults within 5–7 days. Previous studies have reported the entomopathogenic activity of GHA (BotaniGard 22 WP) and NI-8 against different pest species, including *L. lineolaris* [37,38,39,40]. As previously reported, the *B. bassiana* strain GHA was effective against *L. lineolaris* at 10^8^ and 10^9^ concentrations, and NI-8 caused *L. lineolaris* mortality at 10^9^ concentrations. However, the entomopathogenic and endophytic activity of newly isolated *B. bassiana* strain JG-1 has not been tested or previously reported against *L. lineolaris*. JG-1 showed significantly higher mortality of *L. lineolaris* adults at 10^8^ and 10^9^ concentrations compared to other *B. bassiana* strains such as GHA and NI-8. SPE-120 and TAMU-490 showed no entomopathogenicity towards *L. lineolaris* adults. SPE-120 is labeled as a soil and plant enhancer containing *B. bassiana*, though its pathogenicity is not reported against any insect species. TAMU-490 has been reported to influence the host plant selection behavior of *L. hesperus* and *Nezara viridula*, though their pathogenicity is not reported against *L. lineolaris*. We observed no difference in the development of *L. lineolaris* nymphs to adults under greenhouse conditions (Figure 3). However, significantly fewer fifth-instar nymphs were observed on some of the *B. bassiana* containing endophytic plants under field conditions.

Endophytes can mediate herbivore–plant interactions by altering the plant volatiles and by producing alkaloid-based defensive compounds in the plant tissues [41,42] or through altering the nutritional quality of plants [43,44]. These alkaloids, flavonoids, and phenolic compounds act as a defense against pathogen infections and are reported to have antibiotic, antiparasitic, and antioxidant activities [45]. The selection of host plants by herbivores may include pre-alighting cues such as plant volatiles, post-alighting cues such as leaf surface chemistry, and mechanoreceptor cues such as trichomes and leaf texture. Sword et al. [32] reported strong negative responses by *L. lineolaris* and *N. viridula* against flower buds and squares of endophytic cotton plants containing *P. inflatum* and *B. bassiana*. We also observed similar negative responses exhibited by *L. lineolaris* males towards *B. bassiana* strains such as SPE-120, GHA, and the new strain JG-1. However, we did not observe any negative response of *L. lineolaris* males or females towards TAMU-490. Also, it was observed that *L. lineolaris* female adults showed a negative response towards *B. bassiana* strains such as JG-1 and NI-8. *B. bassiana* strain JG-1 elicited negative responses from both male and female *L. lineolaris*. Adult emergence was significantly low in JG-1 treatment. The mortality of *Lygus* adults was also higher for JG-1 at the 10^9^ concentration. *B. bassiana* strain JG-1 exhibited the characteristics of an endophytic entomopathogenic fungi by eliciting negative responses from *L. lineolaris*, and by causing high mortality of *L. lineolaris* adults in the mortality assays.

Endophytes have been reported to improve drought tolerance and reduce attacks by pests, thereby increasing overall crop yield. Two patent claims have been reported on the use of fungal endophytes that show resistance to drought, cold, salt, fungi, bacteria, and pests in cotton (*Dothideomycetes* spp.) and show improved tolerance to drought and pests [32,46]. Greenhouse studies showed a higher number of squares produced by endophytic cotton plants containing *B. bassiana* strains, such as SPE-120 and NI-8, compared to the control. In our field studies, we did not observe any significant differences in cotton yield between the different fungal endophytes studied. However, the yield was noticeably higher in some of the *B. bassiana* treated strains such as NI-8, GHA, and SPE-120, but was not statistically significant (Figure 7).

Laboratory and greenhouse assays clearly showed the establishment of fungal endophytes on cotton plants and their presence in different plant tissues, including fruiting sites. The presence of endophytes in cotton squares resulted in negative responses from *L. lineolaris* adults (Figure 4). Also, it caused developmental delays in *L. lineolaris* nymphs (Figure 6C). Very few early-instar *L. lineolaris* nymphs were observed on NI-8-, GHA-, JG-1-, and SPE-120-treated endophytic cotton plants compared to untreated cotton under field conditions (Figure 5A). However, these did not translate to differences in cotton yield. There could be multiple biotic and abiotic factors that could influence the yield parameters under field conditions. Gehring et al. [47] reported that mycorrhizal fungal associations result in increased stress tolerance, and the adaptive capabilities of the plant increased substantially [48]. Changes in plant volatile profile following endophyte colonization may play an important role in repelling herbivores and reducing plant damage. Sword et al. [32] reported differences in the host selection behavior of *L. hesperus* towards endophytic cotton squares containing *P. inflatum* and *B. bassiana*. However, later studies using solid-phase micro-extraction (SPME) showed no significant differences in the leaf volatile emission profile of these endophytic plants [49]. Multiple factors associated with colonization and persistence of endophytes in plants and their co-existence with native plant microbiomes may influence the establishment and performance of these endophytes. Further research is required to understand these interactions between fungal endophytes and cotton plants, and how they affect host plant resistance and plant growth characteristics.

## 4. Materials and Methods

### 4.1. Fungal Endophytes

Fungal endophytes were selected based on previous research studies and new strains identified in our research. The fungal endophyte *Phialemonium inflatum* (=*Paecilomyces inflatus*) (strain TAMU-490) was originally isolated from surface-sterilized cotton leaves as part of a survey of naturally occurring foliar fungal endophytes in College Station, TX, USA. A culture of TAMU-490 was received from Sword’s lab (Texas A&M, College Station, TX, USA). SPE-120 is a soil and plant enhancer containing *B. bassiana* that forms a symbiotic relationship with plants (Jabb Incorporated in Raleigh, NC, USA). SPE-120 is applied as an inoculant that becomes a symbiotic endophyte in the plant and improves plant health, quality and yield in soybeans, corn, potato, and wheat. JG-1 is a *B. bassiana* strain isolated locally from corn plants in Stoneville, MS, USA. Another endophyte used for comparison was the GHA strain of *B. bassiana* (BotaniGard 22 WP), a commercial mycoinsecticide approved by EPA for use against many insect pests. NI-8, an isolate of *B. bassiana* naturally infecting tarnished plant bug in the Mississippi Delta, was also tested to study its efficacy as an endophyte in cotton against tarnished plant bugs. Endophytes TAMU-490, NI-8, GS-1, and JG-1 were previously isolated from multiyear field surveys of naturally occurring fungal endophytes in cotton and corn [50,51]. An endomycorrhizal fungi containing *Rhizophagous intraradices* (300 propagules/gm) (Wallace organic Wonder, Greene, RI, USA) was also used as a comparison in some of the studies to understand its effects on *L. lineolaris* and cotton yield. All the fungal endophytes were cultured, and spores were used to inoculate untreated cotton seeds.

### 4.2. Spore Culturing and Seed Preparation

Spore culturing: Spore powder production was accomplished by utilizing a biphasic culture system, as described by Portilla et al. [52] and Glover et al. [53]. Harvested technical powder was analyzed for conidia concentration (spores per milliliter) and germination. Fresh potato dextrose agar plates were utilized to extract fungal plugs from 3-day old hyphal growth, which were then suspended in 1000 mL of potato dextrose broth. This mixture was agitated for seven days at 27 °C in an incubator shaker (Excella E25, New Brunswick Scientific Co., Inc., Edison, NJ, USA) to create the inoculum for bioassays. Plastic bags (560 mm × 385 mm × 225 mm) containing 1000 g of white rice and 600 mL of water were autoclaved and inoculated with 150 mL aliquots of the prepared inoculum. The bags were kept in an environmental chamber (27 °C, 50% RH, dark 0/24 h L/D photoperiod) for seven to ten days to allow for full colonization, with the bags being flipped every 24 h. The colonized rice was dried in paper bags (30 cm × 17 cm × 43 cm Barrel, Kraft, Chicago, IL, USA) for seven to ten days until the moisture content was sufficiently low (aw ≤ 0.3). Conidia were manually separated from the dried rice using graded sieves (Grainger, Sieve SS Frame 8, SS Mesh # 15, 30, and 100). The harvested spores were plated on PDA, incubated for 24 h to assess germination, and counted (spores mm^2^). The produced spores were then stored at −80 °C at the Southern Insect Management Research Unit (SIMRU) in Stoneville, MS, USA.

Seed preparation: Seeds were surface-sterilized by immersion in 70% ethanol for 3 min with constant stirring using a magnetic stir bar, immediately followed by 3 min in 2% sodium hypochlorite (NaOCl), followed by three washes in sterile water, based on Lopez et al. [21]. Wash water was plated on PDA media to confirm surface sterilization efficiency. The seeds were then coated with one concentration of the four fungi and two commercial products, in addition to sterile water used as the control. Spore concentrations for each fungus were zero (water control), and ≥*n* × 10^7^ spores/mL based on concentrations used in previous studies of endophytic entomopathogens [21,54]. A hemocytometer 0.1 mm deep (Hausser-Bright Line, Horsham, PA, USA) was used to calculate the conidia concentrations of the resulting stock solutions from diluting 1.0 g of harvested spores containing 6.1 × 10^11^ spores suspended in 50 mL of 0.04% Tween-80 (P8074, Sigma-Aldrich, St. Louis, MO, USA) and methyl cellulose (M7027, Sigma-Aldrich, St. Louis, MO, USA) 2% *v*/*v*. Aliquots of 2 mL suspensions (*n* × 10^7^) provided a concentration of ≥550 ± 25 viable spores per mm^2^ with 500 ± 15 viable spores (95% viability) for all treatments. Fungal-endophyte-coated seeds were dried under a forced air fan on aluminum foil for 12 h and planted within 24 h of the experimental coating application.

Bollgard II™ (DP1646, Delta and Pine Land Company™, Scott, MS, USA) seeds were planted in a potting mix containing 1:2 proportion of potting mix/topsoil. Four sets of ten plants each were planted for each treatment for a total of 40 plants/treatment. The plants were kept under greenhouse conditions under a 16:8 light/dark cycle. The plants were watered regularly and fertilized biweekly. Samples were collected from stems, leaves, squares, and roots 45 days after planting to check for the presence of fungal endophytes by culturing them on an agar medium. Following sporulation, samples of the fungal spores and hyphae were collected and analyzed using PCR.

Plant growth measurements were performed during different growth stages of cotton, including seed germination (5 days after planting), number of true leaves (14 days after germination), plant height (35 days after germination), number of squares (35 days after germination), and dry weights of roots, stem, and whole plant. Roots were cut 7.5 cm above the bottom tip of the taproot. After cutting the roots, they were thoroughly washed using tap water to remove all soil and placed into labeled brown paper bags. The remaining plant stem and leaves were transferred to another brown paper bag. The paper bags were put into an oven at 70 °C for 20 h. After all of the roots and stems were dried, they were weighed on a scale, and dry weight was recorded. Data were analyzed by ANOVA followed by Tukey’s HSD for mean comparisons using JMP statistical software (v. 10, SAS Inc, Cary, NC, USA). Treatments that have no letters in common within the row were significantly different (α = 0.05).

### 4.3. Detection and Validation of Endophytic Fungal Species in Treated Plants

Pieces of cotton square tissue from plants inoculated with *B. bassiana* strains JG1, GHA, SPE and, *P. inflatum* were surface-sterilized with 0.5% sodium hypochlorite solution for 2 min and washed with sterile distilled water three times, followed by three washes with 75% ethanol under a laminar flow sterile hood. Small pieces of surface-sterilized square tissue were cut with a sterile razor blade. One set of square tissue from each plant was placed in 115 mm Petri dishes containing PDA-agar medium to facilitate the growth of endophytes and another set was frozen at −80 °C in sterile 1.5 mL centrifuge tubes. Genomic DNA was extracted from the fungal cultures resulting from plant tissues using MasterPure tissue and cell lysis reagent kit (Epicentre Technologies, Madison, WI, USA) following the manufacturer’s protocol as outlined in the work of Perera et al. [55]. Briefly, two sterile stainless steel ball bearings (3 mm) were placed in each tube containing plant or fungal hyphae and homogenized for 2 min using a bead beater (Biospec Products, Bartlesville, OK, USA). Proteinase K was added to the homogenate to a final concentration of 2 ng/µL and was incubated at 65 °C for two hours, followed by digestion with RNAse at 37 °C for 30 min and precipitation of proteins with MPC solution by incubating on ice for 30 min. The tubes were centrifuged at 16,000× *g* for 15 min at 4 °C, and the supernatant containing DNA was transferred to new tubes. An equal volume of 100% isopropanol was added to the supernatant, mixed well, and the DNA was precipitated by centrifuging at 16,000× *g* for 15 min. DNA pellets were rinsed three times with 70% ethanol, air dried, and resuspended in 35 µL of Tris-HCl, pH 7.5. Genomic DNA was also extracted using the above protocol from the parent fungal stocks used to inoculate cotton plants.

Internal transcribed spacers 1 (ITS1). 5.8S rRNA and ITS2 of the ribosomal RNA gene were PCR-amplified from each DNA sample using a forward primer designed for 18S rRNA (4046_18SF: 5′-CGCTACTACCGATTGAATGGCTC-3′) and a reverse primer for 28S rRNA (4053_28SR: 5′-TCCTCCGCTTATTGATATGC-3′). Standard Taq polymerase and 1× buffer (New England Biolabs, Ipswich, MA, USA) was used on a PTC-200 thermal cycler with a thermal cycling profile containing 30 s initial denaturation at 95 °C, followed by 35 cycles of 10 s denaturation at 95 °C, 15 s annealing at 52 °C, 60 s extension at 72 °C and a final extension of 5 min at 72 °C. Amplicons were resolved in a 1.0% agarose gel to verify amplification.

Ribosomal RNA amplicons from the samples were cleaned by binding to AmPure XP paramagnetic beads (Beckman Coulter, Indianapolis, IN, USA) at a DNA/beads ratio of 1:1.8. Nucleotide sequences of the purified amplicons were obtained by direct sequencing with Flongle flowcell from Oxford Nanopore sequencing technology (Oxford Nanopore Technologies, New York, NY, USA) using the library construction reagent set SQK-LSK114 and the native DNA barcode kit SQK-NDB-96. Nucleotide sequences were analyzed using CLC Genome WorkBench v22.02 (Qiagen, Redwood City, CA, USA).

Nucleotide sequences generated from fungal stocks and published ITS sequences from *B. bassiana* (AB576868, MG548313, and LC768985), and *P. inflatum* (MH857776) were used as references to map the ITS1/2 sequence reads produced from fungal cultures obtained from cotton square tissues. Mismatch cost of 2, linear gap cost of 3 for insertions and deletions, length fraction of 0.90, and similarity fraction of 0.96 were used in the mapping of Oxford Nanopore sequences to the reference sequences. Phylogenetic analysis was performed using Molecular Evolutionary Genetic Analysis (MEGA) 11 [56] based on a multiple-sequence alignment of the ITS1/2 regions. Gene phylogenetic relationships were estimated using the Maximum Likelihood (ML) method [57,58] applying the Kimura-2-Parameter model of sequence evolution [59], that implemented a discrete Gamma shape parameter and node support inferred from 10,000 bootstrap pseudo replications [60] of the aligned nucleotide sequences. ITS1/2 nucleotide sequences of *B. bassiana* and *P. inflatum* obtained from the NCBI database were also used as references. *Cephalotheca sulfurea* ITS sequence (AB278194) was used as the outgroup.

### 4.4. Lygus lineolaris Mortality Following Direct Exposure to Fungal Spores

Four-day-old, tarnished plant bug adults were exposed to serial dilutions of spores from six endophytes at concentrations of (*n* × 10^9^, *n* × 10^8^, *n* × 10^7^). Mortality and sporulation were monitored daily over a 10-d period. The bioassays were carried out in an environmental chamber (Percival Scientific, Perry, IA, USA) set at 28 °C, with a photoperiod of 16:8 h (L:D) and 65% RH. Each treatment (endophyte concentration × fungal endophyte) was replicated three times, involving a total of 270 adults. Spores harvested from four candidate endophytes (TAMU-490, N-I8, GS-1, JG-1) and two commercial products SPE-120 (SBb-2.5 inoculant) and GHA (BotaniGard 22 WP) were diluted with deionized water to achieve the specified concentrations (*n* × 10^9^, *n* × 10^8^, *n* × 10^7^) and estimated based on dilution of *n* × 10^9^ concentration. The number of spores applied was adjusted for viability across all concentrations tested. Aliquots of 5 mL suspensions (*n* × 10^9^) provided a concentration of ≥450 ± 75 viable spores per mm^2^ with 398 ± 25 viable spores (95% viability) for all endophytes and commercial products tested.

Spore concentrations per milliliter were determined using a hemocytometer (0.1 mm deep, Hausser-Bright Line, Horsham, PA, USA) by diluting 1.0 g of harvested spores, which contained 3.1 × 10^11^ spores in 50 mL of 0.04% Tween-80 (Sigma-Aldrich P8074). Conidia suspensions were sprayed onto disposable glass microscope coverslips (Fisherbrand™, Thermo Fisher Scientific, Waltham, MA, USA) using a handheld sprayer, covering an area of 38.5 mm^2^. Spore concentration (spores per mm^2^) was determined by counting the deposited spores. This process was repeated three times at a final concentration of (*n* × 10^9^), with specific dilutions for each endophyte: 5.1, 1.2, 8.1, 1.3, 6.1, and 9.0 for TAMU-490, NI-8, GS-1, JG-1, SPE-120, and GHA, respectively. Tarnished plant bugs were monitored daily for ten days under a light microscope. Bugs were deemed dead if no movement was observed when prodded with a natural hair paint brush and alive if movement was detected. Bugs remained in their original diet cups, with mycosis checked daily by noting the first appearance of external hyphal growth. Data were analyzed using ANOVA and Tukey’s HSD for mean comparisons with JMP statistical software (v. 10, SAS Inc., Cary, NC, USA), considering treatments significantly different if they had no common letters within the row (α = 0.05)

### 4.5. Survival and Development of Lygus lineolaris Nymphs on Fungal Endophytic Plants

Greenhouse experiments were performed on individual cotton plants. Cotton terminals were isolated with insect enclosure bags constructed from organza (22 × 22 × 8 mm ~240 µm mesh, JoAnn’s Fabrics, Hudson, OH, USA) that enclosed the plant terminals affixed with pipe cleaners (top five nodes) containing many squaring sites. The second week of bloom (≈65 d) was chosen to reflect not only a physiologically vulnerable growth stage of cotton but also a location that is commonly associated with economic damage from tarnished plant bug. Nymphal development of *L. lineolaris* was measured by introducing second-instar nymphs to different fungal endophytic plants. Ten second-instar *L. lineolaris* nymphs were introduced to the terminal of each plant with multiple developing squares in a mesh bag, and a total of ten plants were used for each treatment. Adult emergence was monitored starting 5 days after introducing the nymphs, and the total number of adults that emerged after 10 days is reported. Data were analyzed by ANOVA followed by Tukey’s HSD for mean comparisons using JMP statistical software (v. 10, SAS Inc., Cary, NC, USA).

### 4.6. Olfactometer Studies Using Lygus lineolaris Adults Towards Endophytic Cotton Squares

Olfactometer studies were performed to study the behavior of male and female *L. lineolaris* towards endophytic cotton squares. Twenty male and twenty female *L. lineolaris* were used in each olfactometer experiment, and a total of 40 adults were studied for each endophytic fungus. Charcoal-purified air was pushed through the arms of the olfactometer, and the airflow rate was set to 250 mL/min and checked using an ADM flow meter. The adults were released at the main arm of the olfactometer, and their response to control cotton squares or endophytic cotton squares was recorded after 10 min. If no responses were observed by 10 min, the insect was discarded, and a new insect was used. Responses were recorded from 20 male and 20 female adults. After every 5 insect responses, the position of the olfactometer was switched to avoid preferences. A clean olfactometer was used after every 10 behavioral responses were completed. Experiments were performed under dark conditions, as preliminary studies have shown that *L. lineolaris* responds better to odorants under dark conditions. Behavior preferences of adults in the Y-tube olfactometer were statistically analyzed by the χ^2^ test of independence (α = 0.05) in JMP (v. 10, SAS Inc., Cary, NC, USA) that gave 95% confidence intervals.

### 4.7. Field Planting, Yield Measurements, and Performance of Fungal Endophytes against Tarnished Plant Bugs and Bollworms

The field experiment was conducted in the summer of 2023 at the Southern Insect Management Research Unit (SIMRU) research farm near Stoneville, MS, USA. The experiment was laid out in a randomized complete block design with three replications. Each plot consisted of 4 rows (101.6 cm in width) approximately 14 m in length. Bollgard II™ (DP1646, Delta and Pine Land Company^TM^, Scott, MS, USA) cotton seed was planted in early June on 91 m rows and 96 cm row centers at a field site of ≈2.1 ha, resulting in a plant stand of ≈106,250 plants/ha (42,500 plants/acre). Ad hoc applications of herbicides and a plant growth regulator (mepiquat chloride, Loveland Products, Inc., Morgantown, KY, USA) were applied equally to all plots in the study. Transform insecticide (Sulfoxaflor, Corteva Agriscience, Indianapolis, IN, USA), Diamond insecticide (Novaluron, Makhteshim Agan of North America, Inc., Raleigh, NC, USA), and Orthene insecticide (Acephate, AMVAC, Newport Beach, CA, USA) at labeled rates was used ca. every 10 d to maintain the plots pest-free until the first week of bloom. Insecticide applications were discontinued 14 days prior to the first and weekly insect sampling. Insect sampling occurred weekly throughout the six weeks of bloom, and treatment decisions were based on tarnished plant bug densities estimated using a black drop cloth. Mechanical harvest occurred on 11 October 2023 (119 d emergence to harvest).

Treatments included an untreated control (control), endophyte-coated seed only, endophyte spray only, and a seed-coated and sprayed combination. The plots were arranged in a randomized complete block design with four replications. Prior to bloom, all *L. lineolaris* samples were taken with a standard 38 cm sweep net (25 sweeps per plot) once per week. After the first bloom, all plots were sampled with a 0.76 m black drop cloth, with two drops per plot taken weekly. Thresholds for current management practices were based on Mississippi State University Extension Services recommendations of three per 0.76 row m when utilizing a black drop cloth [61]. The efficacy of endophytes was evaluated by taking two drops, using a black drop cloth 6 row feet in length, from the center of two rows of each plot. Total *L. lineolaris* (nymphs and adults) were reported from each plot. *L. lineolaris* were recorded as small (first–third instar) and large (fourth–fifth instar) based on the presence or absence of wing pads and fully developed adults. Cotton bollworm larval stages were pooled. Control plants were sprayed with water. Treatments consisted of treated seeds (coated with spores) of endophyte alone and in combination with a foliar endophyte spray. The plots were sprayed once plot averages reached the threshold for *L. lineolaris*. Data were subject to statistical analysis using one-way ANOVA in SAS software (version 9.4, SAS Institute Inc., Cary, NC, USA). All data were analyzed as randomized complete block designs. Each treatment consisted of 4 to 6 replicates. Data were analyzed using the General Linear Model, and means were compared using the Tukey–Kramer HSD test for least squared means (α = 0.05).

## 5. Conclusions

Endophytic entomopathogenic fungi can influence plant growth and the infestation rate of different pests and diseases. Our greenhouse and field experiments showed that these fungal endophytes can colonize different plant parts, impact the plant growth of cotton, and affect the biology and behavior of *L. lineolaris* adults and nymphs. All the different *B. bassiana* strains tested had some effect on the development or mortality of *L. lineolaris*. The new *B. bassiana* strain JG-1 significantly affected the olfactory response of male and female *L. lineolaris* adults and caused significant adult mortality in the bioassays. Volatile collection studies and GC-MS analysis may provide more details of the induced plant volatiles that cause negative responses from *L. lineolaris* adults. Also, further experiments may help to optimize JG-1 as an entomopathogenic endophytic fungi for practical application against different agricultural pests.

## Figures and Tables

**Table 1:** Cotton plant growth parameter measurements following the application of fungal endophytes or arbuscular mycorrhizal fungus as seed application. Plant growth parameters measured included percentage germination, number of true leaves, squares, plant height, and dry weight of roots, stems, and whole plant. Data were analyzed by ANOVA followed by Tukey’s HSD for mean comparisons. Treatments that have no letters in common within the row were significantly different (α = 0.05).

Growth Parameters	Fungal Endophytes							F-Value	*p*-Value
	TAMU 490	GHA	NI-8	JG-1	SPE-120	AMF	Control		
% germination	80	77.5	95	75	70	82.5	77.5	1.44	0.19
True leaf count	3.6 ± 0.13	3.5 ± 0.11	3.6 ± 0.12	3.3 ± 0.15	3.5 ± 0.15	3.2 ± 0.15	3.2 ± 0.14	1.21	0.31
Square count	3.2 ± 0.18 ^bc^	3.0 ± 0.23 ^bc^	3.9 ± 0.20 ^ab^	2.8 ± 0.19 ^c^	4.2 ± 0.20 ^a^	3.2 ± 0.19 ^abc^	2.4 ± 0.24 ^c^	6.64	<0.0001
Plant height (cm)	20.8 ± 0.86 ^b^	26.9 ± 0.52 ^a^	27.0 ± 0.66 ^a^	25.2 ± 0.48 ^a^	25.4 ± 0.31 ^a^	27.1 ± 0.53 ^a^	25.5 ± 0.53 ^a^	11.92	<0.0001
Root dry weight (gm)	6.8 ± 0.50	6.8 ± 0.71	7.5 ± 0.47	6.1 ± 0.43	7.0 ± 0.55	6.8 ± 0.51	5.4 ± 0.46	1.66	0.14
Stem dry weight (gm)	23.6 ± 1.00 ^b^	21.6 ± 1.70 ^bc^	26.6 ± 1.48 ^ab^	23.4 ± 0.77 ^b^	17.3 ± 1.08 ^c^	30.5 ± 1.98 ^a^	17.3 ± 1.11 ^c^	12.10	<0.0001
Whole plant dry weight (gm)	30.4 ± 1.02 ^bc^	28.4 ± 1.83 ^bcd^	34.1 ± 1.71 ^ab^	29.5 ± 0.73 ^bc^	24.3 ± 1.33 ^cd^	37.3 ± 2.31 ^a^	22.7 ± 1.23 ^d^	11.12	<0.0001

## Data Availability

Data is contained within the article or Supplementary Material.

## Supplementary Materials

The following supporting information can be downloaded at: https://www.mdpi.com/article/10.3390/plants13152012/s1.

## References

[1] Hardoim P.R., Van Overbeek L.S., Berg G., Pirttilä A.M., Company S., Campisano A., Döring M., Sessitsch A. (2015). The hidden world within plants: Ecological and evolutionary considerations for defining functioning of microbial endophytes. Microbiol. Mol. Biol. Rev..

[2] Puri A., Padda K.P., Chanway C.P. (2016). Evidence of nitrogen fixation and growth promotion in canola (*Brassica napus* L.) by an endophytic diazotroph *Paenibacillus polymyxa* P2b-2R. Biol. Fertil. Soils..

[3] Kumar K.K., Dara S.K. (2021). Fungal and bacterial endophytes as microbial control agents for plant-parasitic nematodes. Inter. J. Environ. Res. Public Health.

[4] Suryanarayanan T.S. (2013). Endophyte research: Going beyond isolation and metabolite documentation. Fungal Ecol..

[5] Smith R.J., Pekrul S., Grula E.A. (1981). Requirement for sequential enzymatic activities for penetration of the integument of the corn earworm (*Heliothis zea*). J. Invert. Pathol..

[6] Bamisile B.S., Dash C.K., Akutse K.S., Keppanan R., Afolabi O.G., Hussain M., Qasim M., Wang L. (2018). Prospects of endophytic fungal entomopathogens as biocontrol and plant growth promoting agents: An insight on how artificial inoculation methods affect endophytic colonization of host plants. Microbiol. Res..

[7] Mantzoukas S., Eliopoulos P.A. (2020). Endophytic entomopathogenic fungi: A valuable biological control tool against plant pests. Appl. Sci..

[8] Posada F., Aime M.C., Peterson S.W., Rehner S.A., Vega F.E. (2007). Inoculation of coffee plants with the fungal entomopathogen *Beauveria bassiana* (*Ascomycota: Hypocreales*). Mycol. Res..

[9] Fürnkranz M., Lukesch B., Müller H., Huss H., Grube M., Berg G. (2012). Microbial diversity inside pumpkins: Microhabitat-specific communities display a high antagonistic potential against phytopathogens. Microb. Ecol..

[10] Jaber L.R., Enkerli J. (2016). Effect of seed treatment duration on growth and colonization of *Vicia faba* by endophytic *Beauveria bassiana* and *Metarhizium brunneum*. Biol. Control.

[11] Jaber L.R., Enkerli J. (2017). Fungal entomopathogens as endophytes: Can they promote plant growth?. Biocontrol Sci. Technol..

[12] Lugtenberg B.J., Caradus J.R., Johnson L.J. (2016). Fungal endophytes for sustainable crop production. FEMS Microbiol. Ecol..

[13] Schardl C.L., Leuchtmann A., Spiering M.J. (2004). Symbioses of grasses with seedborne fungal endophytes. Annu. Rev. Plant Biol..

[14] Akutse K.S., Fiaboe K.K., Van den Berg J., Ekesi S., Maniania N.K. (2014). Effects of endophyte colonization of *Vicia faba* (Fabaceae) plants on the life–history of leafminer parasitoids *Phaedrotoma scabriventris* (Hymenoptera: Braconidae) and *Diglyphus isaea* (Hymenoptera: Eulophidae). PLoS ONE.

[15] Saikkonen K., Gundel P.E., Helander M. (2013). Chemical ecology mediated by fungal endophytes in grasses. J. Chem. Ecol..

[16] White J.F., Belanger F.A., Meyer W.I., Sullivan R.F., Bischoff J.F., Lewis E.A. (2002). Minireview article: Clavicipitalean fungal epibionts and endophytes-development of symbiotic interactions with plants. Symbiosis.

[17] Hu S., Bidochka M.J. (2021). Root colonization by endophytic insect-pathogenic fungi. J. Appl. Microbiol..

[18] Arnold A.E., Lewis L.C. (2005). Ecology and evolution of fungal endophytes, and their roles against insects. Insect-Fungal Associations: Ecology and Evolution.

[19] Reddy N.P., Khan A.P., Devi U.K., Sharma H.C., Reineke A. (2009). Treatment of millet crop plant (*Sorghum bicolor*) with the entomopathogenic fungus (*Beauveria bassiana*) to combat infestation by the stem borer, *Chilo partellus* Swinhoe (Lepidoptera: Pyralidae). J. Asia-Pacific Entomol..

[20] Lopez C.D., Zhu-Salzman K., Ek-Ramos M.J., Sword G.A. (2014). The entomopathogenic fungal endophytes *Purpureocillium lilacinum* (formerly *Paecilomyces lilacinus*) and *Beauveria bassiana* negatively affect cotton aphid reproduction under both greenhouse and field conditions. PLoS ONE.

[21] Lopez D.C., Sword G.A. (2015). The endophytic fungal entomopathogens *Beauveria bassiana* and *Purpureocillium lilacinum* enhance the growth of cultivated cotton (*Gossypium hirsutum*) and negatively affect survival of the cotton bollworm (*Helicoverpa zea*). Biol. Control.

[22] Leckie B.M., Ownley B.H., Pereira R.M., Klingeman W.E., Jones C.J., Gwinn K.D. (2008). Mycelia and spent fermentation broth of *Beauveria bassiana* incorporated into synthetic diets affect mortality, growth, and development of larval *Helicoverpa zea* (Lepidoptera: Noctuidae). Biocontrol Sci. Technol..

[23] Menjivar R.D., Cabrera J.A., Kranz J., Sikora R.A. (2012). Induction of metabolite organic compounds by mutualistic endophytic fungi to reduce the greenhouse whitefly *Trialeurodes vaporariorum* (Westwood) infection on tomato. Plant Soil.

[24] Akutse K.S., Maniania N.K., Fiaboe K.K., Van den Berg J., Ekesi S.J. (2013). Endophytic colonization of *Vicia faba* and *Phaseolus vulgaris* (Fabaceae) by fungal pathogens and their effects on the life-history parameters of *Liriomyza huidobrensis* (Diptera: Agromyzidae). Fungal Ecol..

[25] Newcombe G., Shipunov A., Eigenbrode S.D., Raghavendra A.K., Ding H., Anderson C.L., Menjivar R., Crawford M., Schwarzländer M. (2009). Endophytes influence protection and growth of an invasive plant. Commun. Integr. Biol..

[26] Biswas C., Dey P., Satpathy S., Satya P., Mahapatra B.S. (2013). Endophytic colonization of white jute (*Corchorus capsularis*) plants by different *Beauveria bassiana* strains for managing stem weevil (*Apion corchori*). Phytoparasitica.

[27] Thakur A., Kaur S., Kaur A., Singh V. (2013). Enhanced resistance to *Spodoptera litura* in endophyte infected cauliflower plants. Environ. Entomol..

[28] Zhou W., Starr J.L., Krumm J.L., Sword G.A. (2016). The fungal endophyte *Chaetomium globosum* negatively affects both above-and belowground herbivores in cotton. FEMS Microbiol. Ecol..

[29] Zhou W., Wheeler T.A., Starr J.L., Valencia C.U., Sword G.A. (2018). A fungal endophyte defensive symbiosis affects plant-nematode interactions in cotton. Plant Soil.

[30] Gurulingappa P., Sword G.A., Murdoch G., McGee P.A. (2010). Colonization of crop plants by fungal entomopathogens and their effects on two insect pests when in planta. Biol. Control.

[31] Quesada-Moraga E., Munoz-Ledesma F.J., Santiago-Alvarez C. (2009). Systemic protection of *Papaver somniferum* L. against *Iraella luteipes* (Hymenoptera: Cynipidae) by an endophytic strain of *Beauveria bassiana* (*Ascomycota: Hypocreales*). Environ. Entomol..

[32] Sword G.A., Tessnow A., Ek-Ramos M.J. (2017). Endophytic fungi alter sucking bug responses to cotton reproductive structures. Insect Sci..

[33] George J., Glover J.P., Gore J., Crow W.D., Reddy G.V.P. (2021). Biology, ecology, and pest management of the tarnished plant bug, *Lygus lineolaris* (Palisot de Beauvois) in southern row crops. Insects.

[34] Nishi O., Sushida H., Higashi Y., Iida Y. (2021). Epiphytic and endophytic colonisation of tomato plants by the entomopathogenic fungus *Beauveria bassiana* strain GHA. Mycology.

[35] Bowles T.M., Barrios-Masias F.H., Carlisle E.A., Cavagnaro T.R., Jackson L.E. (2016). Effects of arbuscular mycorrhizae on tomato yield, nutrient uptake, water relations, and soil carbon dynamics under deficit irrigation in field conditions. Sci. Total Environ..

[36] Rouphael Y., Franken P., Schneider C., Schwarz D., Giovannetti M., Agnolucci M., De Pascale S., Bonini P., Colla G. (2015). Arbuscular mycorrhizal fungi act as biostimulants in horticultural crops. Sci. Hortic..

[37] Portilla M., Snodgrass G., Luttrell R. Effects of morning and night applications of Beauveria. bassiana strains NI8 and GHA against the tarnished plant bug in cotton. Proceedings of the Beltwide Cotton Conference.

[38] Gad A.A., Nada M.S. (2020). Effect of entomopathogenic fungi *Beauveria bassiana* on the cellular immunity and biochemistry of green bug *Nezara viridula* L.. J. Biopest..

[39] Lopes R.B., Laumann R.A., Blassioli-Moraes M.C., Borges M., Faria M. (2015). The fungistatic and fungicidal effects of volatiles from metathoracic glands of soybean-attacking stink bugs (Heteroptera: Pentatomidae) on the entomopathogen *Beauveria bassiana*. J. Invertebr. Pathol..

[40] Sosa-Gómez D.R., Moscardi F. (1998). Laboratory and field studies on the infection of stink bugs, *Nezara viridula*, *Piezodorus guildinii*, and *Euschistus heros* (Hemiptera: Pentatomidae) with *Metarhizium anisopliae* and *Beauveria bassiana* in Brazil. J. Invertebr. Pathol..

[41] Clay K., Holah J. (1999). Fungal endophyte symbiosis and plant diversity in successional fields. Science.

[42] Faeth S.H. (2002). Are endophytic fungi defensive plant mutualists?. Oikos.

[43] Bernays E.A. (2017). Plant sterols and host-plant affiliations of herbivores. Insect-Plant Interact. (1992).

[44] Jallow M.F., Dugassa-Gobena D., Vidal S. (2008). Influence of an endophytic fungus on host plant selection by a polyphagous moth via volatile spectrum changes. Arthropod Plant Interact..

[45] Strobel G.A. (2003). Endophytes as sources of bioactive products. Microbes Infect..

[46] Chitnis V.R., Suryanarayanan T.S., Nataraja K.N., Prasad S.R., Oelmüller R., Shaanker R.U. (2020). Fungal endophyte-mediated crop improvement: The way ahead. Front. Plant Sci..

[47] Gehring C.A., Sthultz C.M., Flores-Rentería L., Whipple A.V., Whitham T.G. (2017). Tree genetics defines fungal partner communities that may confer drought tolerance. Proc. Natl. Acad. Sci. USA.

[48] Lau J.A., Lennon J.T., Heath K.D. (2017). Trees harness the power of microbes to survive climate change. Proc. Natl. Acad. Sci. USA.

[49] Gale C.C., Suh C.P., Perez J., Lesne P., Wilson C., Kramer Z., Madamba C., Sword G.A. (2021). Sampling volatile organic compounds from individual cotton leaves to test effects of fungal endophyte treatments. Southwest. Entomol..

[50] Ek-Ramos M.J., Zhou W., Valencia C.U., Antwi J.B., Kalns L.L., Morgan G.D., Kerns D.L., Sword G.A. (2013). Spatial and temporal variation in fungal endophyte communities isolated from cultivated cotton (*Gossypium hirsutum*). PLoS ONE.

[51] McGuire M.R., Leland J.E., Dara S., Park Y.H., Ulloa M. (2006). Effect of different isolates of *Beauveria bassiana* on field populations of *Lygus hesperus*. Biol. Control.

[52] Portilla M., Jones W., Perera O., Seiter N., Greene J., Luttrell R. (2016). Estimation of median lethal concentration of three isolates of *Beauveria bassiana* for control of *Megacopta cribraria* (Heteroptera: Plataspidae) bioassayed on solid *Lygus* spp. diet. Insects.

[53] Glover J.P., Nufer M.I., Perera O.P., Portilla M., George J. (2023). Entomopathogenicity of Ascomycete fungus *Cordyceps militaris* on the cotton bollworm, *Helicoverpa zea* (Boddie) (Lepidoptera: Noctuidae). J. Fungi..

[54] Gurulingappa P., McGee P.A., Sword G. (2011). Endophytic *Lecanicillium lecanii* and *Beauveria bassiana* reduce the survival and fecundity of *Aphis gossypii* following contact with conidia and secondary metabolites. Crop. Prot..

[55] Perera O.P., Little N.S., Abdelgaffar H., Jurat-Fuentes J.L., Reddy G.V. (2021). Genetic knockouts indicate that the ABCC2 protein in the bollworm *Helicoverpa zea* is not a major receptor for the Cry1Ac insecticidal protein. Genes.

[56] Tamura K., Stecher G., Kumar S. (2021). MEGA11: Molecular evolutionary genetics analysis version 11. Mol. Biol. Evol..

[57] Nei M., Kumar S. (2000). Molecular Evolution and Phylogenetics.

[58] Beerli P., Felsenstein J. (2001). Maximum likelihood estimation of a migration matrix and effective population sizes in n subpopulations by using a coalescent approach. Proc. Natl. Acad. Sci. USA.

[59] Kimura M. (1980). A simple method for estimating evolutionary rates of base substitutions through comparative studies of nucleotide sequences. J. Mol. Evol..

[60] Felsenstein J. (1985). Confidence limits on phylogenies: An approach using the bootstrap. Evolution.

[61] Catchot A.L., Crow W.D., Dodds D., Gore J., Musser F.R., Irby T., Cook D.R., Layton M.B., Larson E. (2020). Insect Control Guide for Agronomic Crops.

