# Supplementary material for: Role of Endophytic Entomopathogenic Fungi in Mediating Host Selection, Biology, Behavior, and Management of Tarnished Plant Bug, Lygus lineolaris (Hemiptera: Miridae)
Source: Plants (Basel). 2024 Jul 23;13(15):2012. doi: 10.3390/plants13152012 (PMC11313774; doi:10.3390/plants13152012)
Supplement: Supplementary file 1 [file plants-13-02012-s001.zip › plants-3092536-supplementary.pdf]

```
                              1                                                                              80
P. inflatum MH857776     (1) ----------------ATGGCTCAGTGAGGCTTTCGGACTGTCCCAGAGAGGTTGGCAACGACCACTCAGGGCCGGAAAG
   P. inflatum TAMU1     (1) CGCTACTACCGATTGAATGGCTCAGTGAGGCTTTCGGACTGGCCCAGAGAGGTTGGCAACGACCACTCAGGGCCGGAAAG
   P. inflatum TAMU2     (1) CGCTACTACCGATTGAATGGCTCAGTGAGGCTTTCGGACTGGCCCAGAGAGGTTGGCAACGACCACTCAGGGCCGGAAAG
           BC01 TAMU     (1) CGCTACTACCGATTGAATGGCTCAGTGAGGCTTTCGGACTGGCCCAGAGAGGTTGGCAACGACCACTCAGGGCCGGAAAG
           BC02 TAMU     (1) CGCTACTACCGATTGAATGGCTCAGTGAGGCTTTCGGACTGGCCCAGAGAGGTTGGCAACGACCACTCAGGGCCGGAAAG
           BC03 TAMU     (1) CGCTACTACCGATTGAATGGCTCAGTGAGGCTTTCGGACTGGCCCAGAGAGGTTGGCAACGACCACTCAGGGCCGGAAAG
C. sulfuria AB278194     (1) CGCTACTACCGATTGAATGGCTCAGTGAGGCTTTCGGACTGGCCCAGAGAGGTTGGCAACGACCACTCAGGGCCGGAAAG
                              81                                                                            160
P. inflatum MH857776    (65) TTATCCAAACTCTGTCATTTAGAGGAAGTAAAAGTCGTAACAAGGTCTCAGTTGGTGAACCAGCGGAGGGATCATTACAG
   P. inflatum TAMU1    (81) TTATCCAAACTCGGTCATTTAGAGGAAGTAAAAGTCGTAACAAGGTCTCCGTTGGTGAACCAGCGGAGGGATCATTACAG
   P. inflatum TAMU2    (81) TTATCCAAACTCGGTCATTTAGAGGAAGTAAAAGTCGTAACAAGGTCTCCGTTGGTGAACCAGCGGAGGGATCATTACAG
           BC01 TAMU    (81) TTATCCAAACTCGGTCATTTAGAGGAAGTAAAAGTCGTAACAAGGTCTCCGTTGGTGAACCAGCGGAGGGATCATTACAG
           BC02 TAMU    (81) TTATCCAAACTCGGTCATTTAGAGGAAGTAAAAGTCGTAACAAGGTCTCCGTTGGTGAACCAGCGGAGGGATCATTACAG
           BC03 TAMU    (81) TTATCCAAACTCGGTCATTTAGAGGAAGTAAAAGTCGTAACAAGGTCTCCGTTGGTGAACCAGCGGAGGGATCATTACAG
C. sulfuria AB278194    (81) TTATCCAAACTCGGTCATTTAGAGGAAGTAAAAGTCGTAACAAGGTCTCCGTTGGTGAACCAGCGGAGGGATCATTACAG
                              161                                                                           240
P. inflatum MH857776   (145) AGTTTAACGACTCCCAAACCACTGTGAA-CATACCCGTACCGTTGCCTCGGCGGGCGGCCCCAGGGCGGGGCCGCAGCCT
   P. inflatum TAMU1   (161) AGTTTAACGACTCCCAAACCACTGTGAA-CATACCCGTACCGTTGCCTCGGCGGGCGGCCCC-GGGCGGGGCCGCAGCCT
   P. inflatum TAMU2   (161) AGTTTAACGACTCCCAAACCACTGTGAA-CATACCCGTACCGTTGCCTCGGCGGGCGGCCCA-GGGCGGGGCCGCAGCCT
           BC01 TAMU   (161) AGTTTAACGACTCCCAAACCACTGTGAA-CATACCCGTACCGTTGCCTCGGCGGGCGGCCCC-GGGCGGGGCCGCAGCCT
           BC02 TAMU   (161) AGTTTAACGACTCCCAAACCACTGTGAA-CATACCCGTACCGTTGCCTCGGCGGGCGGCCCA-GGGCGGGGCCGCAGCCT
           BC03 TAMU   (161) AGTTTAACGACTCCCAAACCACTGTGAA-CATACCCGTACCGTTGCCTCGGCGGGCGGCCCA-GGGCGGGGCCGCAGCCT
C. sulfuria AB278194   (161) AGTTTAACGACTCCCAAACCACTGTGAAACATACCCGTACCGTTGCCTCGGTGGGCGGCCCCAGGGCGGGGCCGCAGCCT
                              241                                                                           320
P. inflatum MH857776   (224) CCCCAGCGGAGGCGCCCGCCGCAGGTCGCAAAACTATAACTATATTTAGTGGCATCTCTGAGTAACTTCCAAACAATCAA
   P. inflatum TAMU1   (239) CCCCAGCGGAGGCGCCCGCCGCAGGTCGCAAAACTATAACTATATTTAGTGGCATCTCTGAGTAACTTCCAAACAATCAA
   P. inflatum TAMU2   (239) CCCCAGCGGAGGCGCCCGCCGCAGGTCGCAAAACTATAACTATATTTAGTGGCATCTCTGAGTAACTTCCAAACAATCAA
           BC01 TAMU   (239) CCCCAGCGGAGGCGCCCGCCGCAGGTCGCAAAACTATAACTATATTTAGTGGCATCTCTGAGTAACTTCCAAACAATCAA
           BC02 TAMU   (239) CCCCAGCGGAGGCGCCCGCCGCAGGTCGCAAAACTATAACTATATTTAGTGGCATCTCTGAGTAACTTCCAAACAATCAA
           BC03 TAMU   (239) CCCCAGCGGAGGCGCCCGCCGCAGGTCGCAAAACTATAACTATATTTAGTGGCATCTCTGAGTAACTTCCAAACAATCAA
C. sulfuria AB278194   (241) CCT-AGCGGAGGCGCCCGCCGCAGGTCACAAAACTATACCGATATCTAGTGGCATCTCTGAGTAACTTCCAAACAATCAA
                              321                                                                           400
P. inflatum MH857776   (304) AACTTTCAACAACGGATCTCTTGGTTCTGGCATCGATGAAGAACGCAGCGAAATGCGATAAGTAATGTGAATTGCAGAAT
   P. inflatum TAMU1   (319) AACTTTCAACAACGGATCTCTTGGTTCTGGCATCGATGAAGAACGCAGCGAAATGCGATAAGTAATGTGAATTGCAGAAT
   P. inflatum TAMU2   (319) AACTTTCAACAACGGATCTCTTGGTTCTGGCATCGATGAAGAACGCAGCGAAATGCGATAAGTAATGTGAATTGCAGAAT
           BC01 TAMU   (319) AACTTTCAACAACGGATCTCTTGGTTCTGGCATCGATGAAGAACGCAGCGAAATGCGATAAGTAATGTGAATTGCAGAAT
           BC02 TAMU   (319) AACTTTCAACAACGGATCTCTTGGTTCTGGCATCGATGAAGAACGCAGCGAAATGCGATAAGTAATGTGAATTGCAGAAT
           BC03 TAMU   (319) AACTTTCAACAACGGATCTCTTGGTTCTGGCATCGATGAAGAACGCAGCGAAATGCGATAAGTAATGTGAATTGCAGAAT
C. sulfuria AB278194   (320) AACTTTCAACAACGGATCTCTTGGTTCTGGCATCGATGAAGAACGCAGCGAAATGCGATAAGTAATGTGAATTGCAGAAT
                              401                                                                           480
P. inflatum MH857776   (384) TCAGTGAATCATCGAATCTTTGAACGCACATTGCGCCCGCCAGCATTCTGGCGGGCATGCCTGTCCGAGCGTCATTTCAA
   P. inflatum TAMU1   (399) TCAGTGAATCATCGAATCTTTGAACGCACATTGCGCCCGCCAGCATTCTGGCGGGCATGCCTGTCCGAGCGTCATTTCAA
   P. inflatum TAMU2   (399) TCAGTGAATCATCGAATCTTTGAACGCACATTGCGCCCGCCAGCATTCTGGCGGGCATGCCTGTCCGAGCGTCATTTCAA
           BC01 TAMU   (399) TCAGTGAATCATCGAATCTTTGAACGCACATTGCGCCCGCCAGCATTCTGGCGGGCATGCCTGTCCGAGCGTCATTTCAA
           BC02 TAMU   (399) TCAGTGAATCATCGAATCTTTGAACGCACATTGCGCCCGCCAGCATTCTGGCGGGCATGCCTGTCCGAGCGTCATTTCAA
           BC03 TAMU   (399) TCAGTGAATCATCGAATCTTTGAACGCACATTGCGCCCGCCAGCATTCTGGCGGGCATGCCTGTCCGAGCGTCATTTCAA
C. sulfuria AB278194   (400) TCAGTGAATCATCGAATCTTTGAACGCACATTGCGCCCGCCAGCATTCTGGCGGGCATGCCTGTCCGAGCGTCATTTCAA
                              481                                                                           560
P. inflatum MH857776   (464) CCCTCAAGCCCTGCTTGGTGTTGGGGCACTACGCGCGAGCGT--AGGCCCTCAAAATCAGTGGCGGACCCGCTGGAGGTC
   P. inflatum TAMU1   (479) CCCTCAAGCCCTGCTTGGTGTTGGGGCACTACGCGCGAGCGT--AGGCCCTCAAAATCAGTGGCGGACCCGCTGGAGGTC
   P. inflatum TAMU2   (479) CCCTCAAGCCCTGCTTGGTGTTGGGGCACTACGCGCGAGCGT--AGGCCCTCAAAATCAGTGGCGGACCCGCTGGAGGTC
           BC01 TAMU   (479) CCCTCAAGCCCTGCTTGGTGTTGGGGCACTACGCGCGAGCGT--AGGCCCTCAAAATCAGTGGCGGACCCGCTGGAGGTC
           BC02 TAMU   (479) CCCTCAAGCCCTGCTTGGTGTTGGGGCACTACGCGCGAGCGT--AGGCCCTCAAAATCAGTGGCGGACCCGCTGGAGGTC
           BC03 TAMU   (479) CCCTCAAGCCCTGCTTGGTGTTGGGGCACTACGCGCGAGCGT--AGGCCCTCAAAATCAGTGGCGGACCCGCTGGAGGTC
C. sulfuria AB278194   (480) CCCTCAAGCCCTGCTTGGTGTTGGGGCACTACGCGCAAGCGTGTAGGCCCTTAAAATCAGTGGCGGACTCGCTGGAGGTC
                              561                                                                           640
P. inflatum MH857776   (542) CGGGCGTAGTAACACATCTCGCCCGAGGTCCCCAGCGTGCCCCTGCCGTTAAACCCCCAAATT-ACAGAAGGT-GACCTC
   P. inflatum TAMU1   (557) CGGGCGTAGTAACACATCTCGCCCGAGGTCCCCAGCGTGCCCCTGCCGTTAAACCCCCAAATTTACAGAAGGTTGACCTC
   P. inflatum TAMU2   (557) CGGGCGTAGTAACACATCTCGCCCGAGGTCCCCAGCGTGCCCCTGCCGTTAAACCCCCAAATTTACAGAAGGTTGACCTC
           BC01 TAMU   (557) CGGGCGTAGTAACACATCTCGCCCGAGGTCCCCAGCGTGCCCCTGCCGTTAAACCCCCAAATTTACAGAAGGTTGACCTC
           BC02 TAMU   (557) CGGGCGTAGTAACACATCTCGCCCGAGGTCCCCAGCGTGCCCCTGCCGTTAAACCCCCAAATTTACAGAAGGTTGACCTC
           BC03 TAMU   (557) CGGGCGTAGTAACACATCTCGCCCGAGGTCCCCAGCGTGCCCCTGCCGTTAAACCCCCAAATTTACAGAAGGTTGACCTC
C. sulfuria AB278194   (560) CGGGCGTAGTAATACATCTCGCCCGAGGTCCCCAGCGTGCCCCTGCCGTTAAACCCCCAAACTTACAGAAGGTTGACCTC
                              641                                               690
P. inflatum MH857776   (620) GGAT----------------------------------------------
   P. inflatum TAMU1   (637) GGATCAGGTAGGAATACCCGCTGAACTTAAGCATATCAATAAGCGGAGGA
   P. inflatum TAMU2   (637) GGATCAGGTAGGAATACCCGCTGAACTTAAGCATATCAATAAGCGGAGGA
           BC01 TAMU   (637) GGATCAGGTAGGAATACCCGCTGAACTTAAGCATATCAATAAGCGGAGGA
           BC02 TAMU   (637) GGATCAGGTAGGAATACCCGCTGAACTTAAGCATATCAATAAGCGGAGGA
           BC03 TAMU   (637) GGATCAGGTAGGAATACCCGCTGAACTTAAGCATATCAATAAGCGGAGGA
C. sulfuria AB278194   (640) GGATCAGGTAGGAATACCCGCTGAACTTAAGCATATCAATAAGCGGAGGA
```

Figure S1a. Alignment of reference sequences of ITS1, 5.8S, and ITS2 regions of ribosomal RNA of *Phialemonium inflatum* (MH857776) were used as reference to generate evolutionary relationships between stock strain TAMU and fungal cultures recovered from cotton squares obtained from plants inoculated with TAMU isolates. *Cephalotheca sulfurea* ITS sequence (AB278194) was used as the outgroup. Nucleotide polymorphisms, insertions, and deletions in the nucleotide sequences are shown in white text with black background.

```
                                  1                                                                              80
B. bassiana AB576868    (1)  CGCTACTACCGATTGAATGGCTCAGTGAGGCGTCCGGACTGGCCCAGGGAGGTGGGCAACTACCCCCCAGGGCCGGAAAG
B. bassiana MG548313    (1)  ----------------------------------------------GGGAGGTGGGCAACTACCCCCCAGGGCCGGAAAG
B. bassiana LC768985    (1)  CGCTACTACCGATTGAATGGCTCAGTGAGGCGTCCGGACTGGCCCAGGGAGGTGGGCAACTACCCCCCAGGGCCGGAAAG
     B. bassiana GHA    (1)  CGCTACTACCGATTGAATGGCTCAGTGAGGCGTCCGGACTGGCCCAGGGAGGTGGGCAACTACCCCCCAGGGCCGGAAAG
            BC10 GHA    (1)  CGCTACTACCGATTGAATGGCTCAGTGAGGCGTCCGGACTGGCCCAGGGAGGTGGGCAACTACCCCCCAGGGCCGGAAAG
            BC11 GHA    (1)  CGCTACTACCGATTGAATGGCTCAGTGAGGCGTCCGGACTGGCCCAGGGAGGTGGGCAACTACCCCCCAGGGCCGGAAAG
            BC12 GHA    (1)  CGCTACTACCGATTGAATGGCTCAGTGAGGCGTCCGGACTGGCCCAGGGAGGTGGGCAACTACCCCCCAGGGCCGGAAAG
     B. bassiana NI8    (1)  CGCTACTACCGATTGAATGGCTCAGTGAGGCGTCCGGACTGGCCCAGGGAGGTGGGCAACTACCCCCCAGGGCCGGAAAG
            BC04 NI8    (1)  CGCTACTACCGATTGAATGGCTCAGTGAGGCGTCCGGACTGGCCCAGGGAGGTGGGCAACTACCCCCCAGGGCCGGAAAG
            BC05 NI8    (1)  CGCTACTACCGATTGAATGGCTCAGTGAGGCGTCCGGACTGGCCCAGGGAGGTGGGCAACTACCCCCCAGGGCCGGAAAG
            BC06 NI8    (1)  CGCTACTACCGATTGAATGGCTCAGTGAGGCGTCCGGACTGGCCCAGGGAGGTGGGCAACTACCCCCCAGGGCCGGAAAG
     B. bassiana JG1    (1)  CGCTACTACCGATTGAATGGCTCAGTGAGGCGTCCGGACTGGCCCAGGGAGGTGGGCAACTACCCCCCAGGGCCGGAAAG
            BC07 JG1    (1)  CGCTACTACCGATTGAATGGCTCAGTGAGGCGTCCGGACTGGCCCAGGGAGGTGGGCAACTACCCCCCAGGGCCGGAAAG
            BC08 JG1    (1)  CGCTACTACCGATTGAATGGCTCAGTGAGGCGTCCGGACTGGCCCAGGGAGGTGGGCAACTACCCCCCAGGGCCGGAAAG
            BC09 JG1    (1)  CGCTACTACCGATTGAATGGCTCAGTGAGGCGTCCGGACTGGCCCAGGGAGGTGGGCAACTACCCCCCAGGGCCGGAAAG
     B. bassiana SPE    (1)  CGCTACTACCGATTGAATGGCTCAGTGAGGCGTCCGGACTGGCCCAGGGAGGTTGGCAACTACCCCC-AGGGCCGGAAAG
            BC17 SPE    (1)  CGCTACTACCGATTGAATGGCTCAGTGAGGCGTCCGGACTGGCCCAGGGAGGTTGGCAACTACCCCC-AGGGCCGGAAAG
            BC18 SPE    (1)  CGCTACTACCGATTGAATGGCTCAGTGAGGCGTCCGGACTGGCCCAGGGAGGTTGGCAACTACCCCC-AGGGCCGGAAAG
                             81                                                                             160
B. bassiana AB576868   (81)  CTCTCCAAACTCGGTCATTTAGAGGAAGTAAAAGTCGTAACAAGGTCTCCGTTGGTGAACCAGCGGAGGGATCATTACCG
B. bassiana MG548313   (35)  CTCTCCAAACTCGGTCATTTAGAGGAAGTAAAAGTCGTAACAAGGTCTCCGTTGGTGAACCAGCGGAGGGATCATTACCG
B. bassiana LC768985   (81)  CTCTCCAAACTCGGTCATTTAGAGGAAGTAAAAGTCGTAACAAGGTCTCCGTTGGTGAACCAGCGGAGGGATCATTACCG
     B. bassiana GHA   (81)  CTCTCCAAACTCGGTCATTTAGAGGAAGTAAAAGTCGTAACAAGGTCTCCGTTGGTGAACCAGCGGAGGGATCATTACCG
            BC10 GHA   (81)  CTCTCCAAACTCGGTCATTTAGAGGAAGTAAAAGTCGTAACAAGGTCTCCGTTGGTGAACCAGCGGAGGGATCATTACCG
            BC11 GHA   (81)  CTCTCCAAACTCGGTCATTTAGAGGAAGTAAAAGTCGTAACAAGGTCTCCGTTGGTGAACCAGCGGAGGGATCATTACCG
            BC12 GHA   (81)  CTCTCCAAACTCGGTCATTTAGAGGAAGTAAAAGTCGTAACAAGGTCTCCGTTGGTGAACCAGCGGAGGGATCATTACCG
     B. bassiana NI8   (81)  CTCTCCAAACTCGGTCATTTAGAGGAAGTAAAAGTCGTAACAAGGTCTCCGTTGGTGAACCAGCGGAGGGATCATTACCG
            BC04 NI8   (81)  CTCTCCAAACTCGGTCATTTAGAGGAAGTAAAAGTCGTAACAAGGTCTCCGTTGGTGAACCAGCGGAGGGATCATTACCG
            BC05 NI8   (81)  CTCTCCAAACTCGGTCATTTAGAGGAAGTAAAAGTCGTAACAAGGTCTCCGTTGGTGAACCAGCGGAGGGATCATTACCG
            BC06 NI8   (81)  CTCTCCAAACTCGGTCATTTAGAGGAAGTAAAAGTCGTAACAAGGTCTCCGTTGGTGAACCAGCGGAGGGATCATTACCG
     B. bassiana JG1   (81)  CTCTCCAAACTCGGTCATTTAGAGGAAGTAAAAGTCGTAACAAGGTCTCCGTTGGTGAACCAGCGGAGGGATCATTACCG
            BC07 JG1   (81)  CTCTCCAAACTCGGTCATTTAGAGGAAGTAAAAGTCGTAACAAGGTCTCCGTTGGTGAACCAGCGGAGGGATCATTACCG
            BC08 JG1   (81)  CTCTCCAAACTCGGTCATTTAGAGGAAGTAAAAGTCGTAACAAGGTCTCCGTTGGTGAACCAGCGGAGGGATCATTACCG
            BC09 JG1   (81)  CTCTCCAAACTCGGTCATTTAGAGGAAGTAAAAGTCGTAACAAGGTCTCCGTTGGTGAACCAGCGGAGGGATCATTACCG
     B. bassiana SPE   (80)  CTCTCCAAACTCGGTCATTTAGAGGAAGTAAAAGTCGTAACAAGGTCTCCGTTGGTGAACCAGCGGAGGGATCATTACCG
            BC17 SPE   (80)  CTCTCCAAACTCGGTCATTTAGAGGAAGTAAAAGTCGTAACAAGGTCTCCGTTGGTGAACCAGCGGAGGGATCATTACCG
            BC18 SPE   (80)  CTCTCCAAACTCGGTCATTTAGAGGAAGTAAAAGTCGTAACAAGGTCTCCGTTGGTGAACCAGCGGAGGGATCATTACCG
                             161                                                                            240
B. bassiana AB576868  (161)  AGTTTTCAACTCCCTAACCCTTCTGTGAACCTACCTATCGTTGCTTCGGCGGACTCGCCCCAGCCCGGACGCGGACTGGA
B. bassiana MG548313  (115)  AGTTTTCAACTCCCTAACCCTTCTGTGAACCTACCTATCGTTGCTTCGGCGGACTCGCCCCAGCCCGGACGCGGACTGGA
B. bassiana LC768985  (161)  AGTTTTCAACTCCCTAACCCTTCTGTGAACCTACCTATCGTTGCTTCGGCGGACTCGCCCCAGCCCGGACGCGGACTGGA
     B. bassiana GHA  (161)  AGTTTTCAACTCCCTAACCCTTCTGTGAACCTACCTATCGTTGCTTCGGCGGACTCGCCCCAGCCCGGACGCGGACTGGA
            BC10 GHA  (161)  AGTTTTCAACTCCCTAACCCTTCTGTGAACCTACCTATCGTTGCTTCGGCGGACTCGCCCCAGCCCGGACGCGGACTGGA
            BC11 GHA  (161)  AGTTTTCAACTCCCTAACCCTTCTGTGAACCTACCTATCGTTGCTTCGGCGGACTCGCCCCAGCCCGGACGCGGACTGGA
            BC12 GHA  (161)  AGTTTTCAACTCCCTAACCCTTCTGTGAACCTACCTATCGTTGCTTCGGCGGACTCGCCCCAGCCCGGACGCGGACTGGA
     B. bassiana NI8  (161)  AGTTTTCAACTCCCTAACCCTTCTGTGAACCTACCTATCGTTGCTTCGGCGGACTCGCCCCAGCCCGGACGCGGACTGGA
            BC04 NI8  (161)  AGTTTTCAACTCCCTAACCCTTCTGTGAACCTACCTATCGTTGCTTCGGCGGACTCGCCCCAGCCCGGACGCGGACTGGA
            BC05 NI8  (161)  AGTTTTCAACTCCCTAACCCTTCTGTGAACCTACCTATCGTTGCTTCGGCGGACTCGCCCCAGCCCGGACGCGGACTGGA
            BC06 NI8  (161)  AGTTTTCAACTCCCTAACCCTTCTGTGAACCTACCTATCGTTGCTTCGGCGGACTCGCCCCAGCCCGGACGCGGACTGGA
     B. bassiana JG1  (161)  AGTTTTCAACTCCCTAACCCTTCTGTGAACCTACCTATCGTTGCTTCGGCGGACTCGCCCCAGCCCGGACGCGGACTGGA
            BC07 JG1  (161)  AGTTTTCAACTCCCTAACCCTTCTGTGAACCTACCTATCGTTGCTTCGGCGGACTCGCCCCAGCCCGGACGCGGACTGGA
            BC08 JG1  (161)  AGTTTTCAACTCCCTAACCCTTCTGTGAACCTACCTATCGTTGCTTCGGCGGACTCGCCCCAGCCCGGACGCGGACTGGA
            BC09 JG1  (161)  AGTTTTCAACTCCCTAACCCTTCTGTGAACCTACCTATCGTTGCTTCGGCGGACTCGCCCCAGCCCGGACGCGGACTGGA
     B. bassiana SPE  (160)  AGTTTTCAACTCCCTAACCCTTCTGTGAACCTACCTATCGTTGCTTCGGCGGACTCGCCCCAGCCCGGACGCGGACTGGA
            BC17 SPE  (160)  AGTTTTCAACTCCCTAACCCTTCTGTGAACCTACCTATCGTTGCTTCGGCGGACTCGCCCCAGCCCGGACGCGGACTGGA
            BC18 SPE  (160)  AGTTTTCAACTCCCTAACCCTTCTGTGAACCTACCTATCGTTGCTTCGGCGGACTCGCCCCAGCCCGGACGCGGACTGGA
                             241                                                                            320
B. bassiana AB576868  (241)  CCAGCGGCCCGCCGGGGACCTCAAACTCTTGTATTCCAGCATCTTCTGAATACGCCGCAAGGCAAAACAAATGAATCAAA
B. bassiana MG548313  (195)  CCAGCGGCCCGCCGGGGACCTCAAACTCTTGTATTCCAGCATCTTCTGAATACGCCGCAAGGCAAAACAAATGAATCAAA
B. bassiana LC768985  (241)  CCAGCGGCCCGCCGGGGACCTCAAACTCTTGTATTCCAGCATCTTCTGAATACGCCGCAAGGCAAAACAAATGAATCAAA
     B. bassiana GHA  (241)  CCAGCGGCCCGCCGGGGACCTCAAACTCTTGTATTCCAGCATCTTCTGAATACGCCGCAAGGCAAAACAAATGAATCAAA
            BC10 GHA  (241)  CCAGCGGCCCGCCGGGGACCTCAAACTCTTGTATTCCAGCATCTTCTGAATACGCCGCAAGGCAAAACAAATGAATCAAA
            BC11 GHA  (241)  CCAGCGGCCCGCCGGGGACCTCAAACTCTTGTATTCCAGCATCTTCTGAATACGCCGCAAGGCAAAACAAATGAATCAAA
            BC12 GHA  (241)  CCAGCGGCCCGCCGGGGACCTCAAACTCTTGTATTCCAGCATCTTCTGAATACGCCGCAAGGCAAAACAAATGAATCAAA
     B. bassiana NI8  (241)  CCAGCGGCCCGCCGGGGACCTCAAACTCTTGTATTCCAGCATCTTCTGAATACGCCGCAAGGCAAAACAAATGAATCAAA
            BC04 NI8  (241)  CCAGCGGCCCGCCGGGGACCTCAAACTCTTGTATTCCAGCATCTTCTGAATACGCCGCAAGGCAAAACAAATGAATCAAA
            BC05 NI8  (241)  CCAGCGGCCCGCCGGGGACCTCAAACTCTTGTATTCCAGCATCTTCTGAATACGCCGCAAGGCAAAACAAATGAATCAAA
            BC06 NI8  (241)  CCAGCGGCCCGCCGGGGACCTCAAACTCTTGTATTCCAGCATCTTCTGAATACGCCGCAAGGCAAAACAAATGAATCAAA
     B. bassiana JG1  (241)  CCAGCGGCCCGCCGGGGACCTCAAACTCTTGTATTCCAGCATCTTCTGAATACGCCGCAAGGCAAAACAAATGAATCAAA
            BC07 JG1  (241)  CCAGCGGCCCGCCGGGGACCTCAAACTCTTGTATTCCAGCATCTTCTGAATACGCCGCAAGGCAAAACAAATGAATCAAA
            BC08 JG1  (241)  CCAGCGGCCCGCCGGGGACCTCAAACTCTTGTATTCCAGCATCTTCTGAATACGCCGCAAGGCAAAACAAATGAATCAAA
            BC09 JG1  (241)  CCAGCGGCCCGCCGGGGACCTCAAACTCTTGTATTCCAGCATCTTCTGAATACGCCGCAAGGCAAAACAAATGAATCAAA
     B. bassiana SPE  (240)  CCAGCGGCCCGCCGGGGACCTCAAACTCTTGTATTCCAGCATCTTCTGAATACGCCGCAAGGCAAAACAAATGAATCAAA
            BC17 SPE  (240)  CCAGCGGCCCGCCGGGGACCTCAAACTCTTGTATTCCAGCATCTTCTGAATACGCCGCAAGGCAAAACAAATGAATCAAA
            BC18 SPE  (240)  CCAGCGGCCCGCCGGGGACCTCAAACTCTTGTATTCCAGCATCTTCTGAATACGCCGCAAGGCAAAACAAATGAATCAAA
```

		321 400
B. bassiana AB576868	(321)	ACTTTCAACAACGGATCTCTTGGCTCTGGCATCGATGAAGAACGCAGCGAAACGCGATAAGTAATGTGAATTGCAGAATC
B. bassiana MG548313	(275)	ACTTTCAACAACGGATCTCTTGGCTCTGGCATCGATGAAGAACGCAGCGAAATGCGATAAGTAATGTGAATTGCAGAATC
B. bassiana LC768985	(321)	ACTTTCAACAACGGATCTCTTGGCTCTGGCATCGATGAAGAACGCAGCGAAATGCGATAAGTAATGTGAATTGCAGAATC
B. bassiana GHA	(321)	ACTTTCAACAACGGATCTCTTGGCTCTGGCATCGATGAAGAACGCAGCGAAACGCGATAAGTAATGTGAATTGCAGAATC
BC10 GHA	(321)	ACTTTCAACAACGGATCTCTTGGCTCTGGCATCGATGAAGAACGCAGCGAAACGCGATAAGTAATGTGAATTGCAGAATC
BC11 GHA	(321)	ACTTTCAACAACGGATCTCTTGGCTCTGGCATCGATGAAGAACGCAGCGAAACGCGATAAGTAATGTGAATTGCAGAATC
BC12 GHA	(321)	ACTTTCAACAACGGATCTCTTGGCTCTGGCATCGATGAAGAACGCAGCGAAACGCGATAAGTAATGTGAATTGCAGAATC
B. bassiana NI8	(321)	ACTTTCAACAACGGATCTCTTGGCTCTGGCATCGATGAAGAACGCAGCGAAATGCGATAAGTAATGTGAATTGCAGAATC
BC04 NI8	(321)	ACTTTCAACAACGGATCTCTTGGCTCTGGCATCGATGAAGAACGCAGCGAAATGCGATAAGTAATGTGAATTGCAGAATC
BC05 NI8	(321)	ACTTTCAACAACGGATCTCTTGGCTCTGGCATCGATGAAGAACGCAGCGAAATGCGATAAGTAATGTGAATTGCAGAATC
BC06 NI8	(321)	ACTTTCAACAACGGATCTCTTGGCTCTGGCATCGATGAAGAACGCAGCGAAATGCGATAAGTAATGTGAATTGCAGAATC
B. bassiana JG1	(321)	ACTTTCAACAACGGATCTCTTGGCTCTGGCATCGATGAAGAACGCAGCGAAATGCGATAAGTAATGTGAATTGCAGAATC
BC07 JG1	(321)	ACTTTCAACAACGGATCTCTTGGCTCTGGCATCGATGAAGAACGCAGCGAAATGCGATAAGTAATGTGAATTGCAGAATC
BC08 JG1	(321)	ACTTTCAACAACGGATCTCTTGGCTCTGGCATCGATGAAGAACGCAGCGAAATGCGATAAGTAATGTGAATTGCAGAATC
BC09 JG1	(321)	ACTTTCAACAACGGATCTCTTGGCTCTGGCATCGATGAAGAACGCAGCGAAATGCGATAAGTAATGTGAATTGCAGAATC
B. bassiana SPE	(320)	ACTTTCAACAACGGATCTCTTGGCTCTGGCATCGATGAAGAACGCAGCGAAACGCGATAAGTAATGTGAATTGCAGAATC
BC17 SPE	(320)	ACTTTCAACAACGGATCTCTTGGCTCTGGCATCGATGAAGAACGCAGCGAAACGCGATAAGTAATGTGAATTGCAGAATC
BC18 SPE	(320)	ACTTTCAACAACGGATCTCTTGGCTCTGGCATCGATGAAGAACGCAGCGAAACGCGATAAGTAATGTGAATTGCAGAATC
		401 480
B. bassiana AB576868	(401)	CAGTGAATCATCGAATCTTTGAACGCACATTGCGCCCGCCAGCATTCTGGCGGGCATGCCTGTTCGAGCGTCATTTCAAC
B. bassiana MG548313	(355)	CAGTGAATCATCGAATCTTTGAACGCACATTGCGCCCGCCAGCATTCTGGCGGGCATGCCTGTTCGAGCGTCATTTCAAC
B. bassiana LC768985	(401)	CAGTGAATCATCGAATCTTTGAACGCACATTGCGCCCGCCAGCATTCTGGCGGGCATGCCTGTTCGAGCGTCATTTCAAC
B. bassiana GHA	(401)	CAGTGAATCATCGAATCTTTGAACGCACATTGCGCCCGCCAGCATTCTGGCGGGCATGCCTGTTCGAGCGTCATTTCAAC
BC10 GHA	(401)	CAGTGAATCATCGAATCTTTGAACGCACATTGCGCCCGCCAGCATTCTGGCGGGCATGCCTGTTCGAGCGTCATTTCAAC
BC11 GHA	(401)	CAGTGAATCATCGAATCTTTGAACGCACATTGCGCCCGCCAGCATTCTGGCGGGCATGCCTGTTCGAGCGTCATTTCAAC
BC12 GHA	(401)	CAGTGAATCATCGAATCTTTGAACGCACATTGCGCCCGCCAGCATTCTGGCGGGCATGCCTGTTCGAGCGTCATTTCAAC
B. bassiana NI8	(401)	CAGTGAATCATCGAATCTTTGAACGCACATTGCGCCCGCCAGCATTCTGGCGGGCATGCCTGTTCGAGCGTCATTTCAAC
BC04 NI8	(401)	CAGTGAATCATCGAATCTTTGAACGCACATTGCGCCCGCCAGCATTCTGGCGGGCATGCCTGTTCGAGCGTCATTTCAAC
BC05 NI8	(401)	CAGTGAATCATCGAATCTTTGAACGCACATTGCGCCCGCCAGCATTCTGGCGGGCATGCCTGTTCGAGCGTCATTTCAAC
BC06 NI8	(401)	CAGTGAATCATCGAATCTTTGAACGCACATTGCGCCCGCCAGCATTCTGGCGGGCATGCCTGTTCGAGCGTCATTTCAAC
B. bassiana JG1	(401)	CAGTGAATCATCGAATCTTTGAACGCACATTGCGCCCGCCAGCATTCTGGCGGGCATGCCTGTTCGAGCGTCATTTCAAC
BC07 JG1	(401)	CAGTGAATCATCGAATCTTTGAACGCACATTGCGCCCGCCAGCATTCTGGCGGGCATGCCTGTTCGAGCGTCATTTCAAC
BC08 JG1	(401)	CAGTGAATCATCGAATCTTTGAACGCACATTGCGCCCGCCAGCATTCTGGCGGGCATGCCTGTTCGAGCGTCATTTCAAC
BC09 JG1	(401)	CAGTGAATCATCGAATCTTTGAACGCACATTGCGCCCGCCAGCATTCTGGCGGGCATGCCTGTTCGAGCGTCATTTCAAC
B. bassiana SPE	(400)	CAGTGAATCATCGAATCTTTGAACGCACATTGCGCCCGCCAGCATTCTGGCGGGCATGCCTGTTCGAGCGTCATTTCAAC
BC17 SPE	(400)	CAGTGAATCATCGAATCTTTGAACGCACATTGCGCCCGCCAGCATTCTGGCGGGCATGCCTGTTCGAGCGTCATTTCAAC
BC18 SPE	(400)	CAGTGAATCATCGAATCTTTGAACGCACATTGCGCCCGCCAGCATTCTGGCGGGCATGCCTGTTCGAGCGTCATTTCAAC
		481 560
B. bassiana AB576868	(481)	CCTCGACCTCCCCTTGGGGGGGTCGGCGTTGGGGACCGGCAGCACACCGCCGGCCCTGAAATGGAGTGGCGGCCCGTCCG
B. bassiana MG548313	(435)	CCTCGACCTCCCCTGGGGGAGGTCGGCGTTGGGGACCGGCAGCACACCGCCGGCCCTGAAATGGAGTGGCGGCCCGTCCG
B. bassiana LC768985	(481)	CCTCGACCTCCCCTTGGGGAGGTCGGCGTTGGGGACCGGCAGCACACCGCCGGCCCTGAAATGGAGTGGCGGCCCGTCCG
B. bassiana GHA	(481)	CCTCGACCTCCCCTTGGGGGGGTCGGCGTTGGGGACCGGCAGCACACCGCCGGCCCTGAAATGGAGTGGCGGCCCGTCCG
BC10 GHA	(481)	CCTCGACCTCCCCTTGGGGGGGTCGGCGTTGGGGACCGGCAGCACACCGCCGGCCCTGAAATGGAGTGGCGGCCCGTCCG
BC11 GHA	(481)	CCTCGACCTCCCCTTGGGGGGGTCGGCGTTGGGGACCGGCAGCACACCGCCGGCCCTGAAATGGAGTGGCGGCCCGTCCG
BC12 GHA	(481)	CCTCGACCTCCCCTTGGGGGGGTCGGCGTTGGGGACCGGCAGCACACCGCCGGCCCTGAAATGGAGTGGCGGCCCGTCCG
B. bassiana NI8	(481)	CCTCGACCTCCCCT-GGGGAGGTCGGCGTTGGGGACCGGCAGCACACCGCCGGCCCTGAAATGGAGTGGCGGCCCGTCCG
BC04 NI8	(481)	CCTCGACCTCCCCT-GGGGAGGTCGGCGTTGGGGACCGGCAGCACACCGCCGGCCCTGAAATGGAGTGGCGGCCCGTCCG
BC05 NI8	(481)	CCTCGACCTCCCCT-GGGGAGGTCGGCGTTGGGGACCGGCAGCACACCGCCGGCCCTGAAATGGAGTGGCGGCCCGTCCG
BC06 NI8	(481)	CCTCGACCTCCCCT-GGGGAGGTCGGCGTTGGGGACCGGCAGCACACCGCCGGCCCTGAAATGGAGTGGCGGCCCGTCCG
B. bassiana JG1	(481)	CCTCCACCTCCCCTGGGGGAGGTCGGCGTTGGGGACCGGCAGCACACCGCCGGCCCTGAAATGGAGTGGCGGCCCGTCCG
BC07 JG1	(481)	CCTCCACCTCCCCTGGGGGAGGTCGGCGTTGGGGACCGGCAGCACACCGCCGGCCCTGAAATGGAGTGGCGGCCCGTCCG
BC08 JG1	(481)	CCTCCACCTCCCCTGGGGGAGGTCGGCGTTGGGGACCGGCAGCACACCGCCGGCCCTGAAATGGAGTGGCGGCCCGTCCG
BC09 JG1	(481)	CCTCCACCTCCCCTGGGGGAGGTCGGCGTTGGGGACCGGCAGCACACCGCCGGCCCTGAAATGGAGTGGCGGCCCGTCCG
B. bassiana SPE	(480)	CCTCGACCTCCCCTTGGGGAGGTCGGCGTTGGGGACCGGCAGCACACCGCCGGCCCTGAAATGGAGTGGCGGCCCGTCCG
BC17 SPE	(480)	CCTCGACCTCCCCTTGGGGAGGTCGGCGTTGGGGACCGGCAGCACACCGCCGGCCCTGAAATGGAGTGGCGGCCCGTCCG
BC18 SPE	(480)	CCTCGACCTCCCCTTGGGGAGGTCGGCGTTGGGGACCGGCAGCACACCGCCGGCCCTGAAATGGAGTGGCGGCCCGTCCG
		561 640
B. bassiana AB576868	(561)	CGGCGACCTCTGCGCAGTAATACAGCTCGCACCGGAACCCCGACGCGGCCACGCCGTAAAACACCCAACTTCTGAACGTT
B. bassiana MG548313	(515)	CGGCGACCTCTGCGTAGTAATACAGCTCGCACCGGAACCCCGACGCGGCCACGCCGTAAAACACCCAACTTCTGAACGTT
B. bassiana LC768985	(561)	CGGCGACCTCTGCGTAGTAATACAGCTCGCACCGGAACCCCGACGCGGCCACGCCGTAAAACACCCAACTTCTGAACGTT
B. bassiana GHA	(561)	CGGCGACCTCTGCGCAGTAATACAGCTCGCACCGGAACCCCGACGCGGCCACGCCGTAAAACACCCAACTTCTGAACGTT
BC10 GHA	(561)	CGGCGACCTCTGCGCAGTAATACAGCTCGCACCGGAACCCCGACGCGGCCACGCCGTAAAACACCCAACTTCTGAACGTT
BC11 GHA	(561)	CGGCGACCTCTGCGCAGTAATACAGCTCGCACCGGAACCCCGACGCGGCCACGCCGTAAAACACCCAACTTCTGAACGTT
BC12 GHA	(561)	CGGCGACCTCTGCGCAGTAATACAGCTCGCACCGGAACCCCGACGCGGCCACGCCGTAAAACACCCAACTTCTGAACGTT
B. bassiana NI8	(560)	CGGCGACCTCTGCGTAGTAATACAGCTCGCACCGGAACCCCGACGCGGCCACGCCGTAAAACACCCAACTTCTGAACGTT
BC04 NI8	(560)	CGGCGACCTCTGCGTAGTAATACAGCTCGCACCGGAACCCCGACGCGGCCACGCCGTAAAACACCCAACTTCTGAACGTT
BC05 NI8	(560)	CGGCGACCTCTGCGTAGTAATACAGCTCGCACCGGAACCCCGACGCGGCCACGCCGTAAAACACCCAACTTCTGAACGTT
BC06 NI8	(560)	CGGCGACCTCTGCGTAGTAATACAGCTCGCACCGGAACCCCGACGCGGCCACGCCGTAAAACACCCAACTTCTGAACGTT
B. bassiana JG1	(561)	CGGCGACCTCTGCGTAGTAATACAGCTCGCACCGGAACCCCGACGCGGCCACGCCGTAAAACACCCAACTTCTGAACGTT
BC07 JG1	(561)	CGGCGACCTCTGCGTAGTAATACAGCTCGCACCGGAACCCCGACGCGGCCACGCCGTAAAACACCCAACTTCTGAACGTT
BC08 JG1	(561)	CGGCGACCTCTGCGTAGTAATACAGCTCGCACCGGAACCCCGACGCGGCCACGCCGTAAAACACCCAACTTCTGAACGTT
BC09 JG1	(561)	CGGCGACCTCTGCGTAGTAATACAGCTCGCACCGGAACCCCGACGCGGCCACGCCGTAAAACACCCAACTTCTGAACGTT
B. bassiana SPE	(560)	CGGCGACCTCTGCGCAGTAATACAGCTCGCACCGGGACCCCGACGCGGCCACGCCGTAAAACACCCAACTTCTGAACGTT
BC17 SPE	(560)	CGGCGACCTCTGCGCAGTAATACAGCTCGCACCGGGACCCCGACGCGGCCACGCCGTAAAACACCCAACTTCTGAACGTT
BC18 SPE	(560)	CGGCGACCTCTGCGCAGTAATACAGCTCGCACCGGGACCCCGACGCGGCCACGCCGTAAAACACCCAACTTCTGAACGTT

```
                                641                                                    696
B. bassiana AB576868  (641) GACCTCGAATCAGGTAGGACTACCCGCTGAACTTAAGCATATCAATAAGCGGAGGA
B. bassiana MG548313  (595) GACCTCGAATCAGGTAGGACTACCCGCTGAACTTAAGCATATCAATAAGCGGAGGA
B. bassiana LC768985  (641) GACCTCGAATCAGGTAGGACTACCCGCTGAACTTAAGCATATCAATAAGCGGAGG-
     B. bassiana GHA  (641) GACCTCGAATCAGGTAGGACTACCCGCTGAACTTAAGCATATCAATAAGCGGAGGA
            BC10 GHA  (641) GACCTCGAATCAGGTAGGACTACCCGCTGAACTTAAGCATATCAATAAGCGGAGGA
            BC11 GHA  (641) GACCTCGAATCAGGTAGGACTACCCGCTGAACTTAAGCATATCAATAAGCGGAGGA
            BC12 GHA  (641) GACCTCGAATCAGGTAGGACTACCCGCTGAACTTAAGCATATCAATAAGCGGAGGA
     B. bassiana NI8  (640) GACCTCGAATCAGGTAGGACTACCCGCTGAACTTAAGCATATCAATAAGCGGAGGA
            BC04 NI8  (640) GACCTCGAATCAGGTAGGACTACCCGCTGAACTTAAGCATATCAATAAGCGGAGGA
            BC05 NI8  (640) GACCTCGAATCAGGTAGGACTACCCGCTGAACTTAAGCATATCAATAAGCGGAGGA
            BC06 NI8  (640) GACCTCGAATCAGGTAGGACTACCCGCTGAACTTAAGCATATCAATAAGCGGAGGA
     B. bassiana JG1  (641) GACCTCGAATCAGGTAGGACTACCCGCTGAACTTAAGCATATCAATAAGCGGAGGA
            BC07 JG1  (641) GACCTCGAATCAGGTAGGACTACCCGCTGAACTTAAGCATATCAATAAGCGGAGGA
            BC08 JG1  (641) GACCTCGAATCAGGTAGGACTACCCGCTGAACTTAAGCATATCAATAAGCGGAGGA
            BC09 JG1  (641) GACCTCGAATCAGGTAGGACTACCCGCTGAACTTAAGCATATCAATAAGCGGAGGA
     B. bassiana SPE  (640) GACCTCGAATCAGGTAGGACTACCCGCTGAACTTAAGCATATCAATAAGCGGAGGA
            BC17 SPE  (640) GACCTCGAATCAGGTAGGACTACCCGCTGAACTTAAGCATATCAATAAGCGGAGGA
            BC18 SPE  (640) GACCTCGAATCAGGTAGGACTACCCGCTGAACTTAAGCATATCAATAAGCGGAGGA
```

Figure S1b. Alignment of reference sequences of ITS1, 5.8S, and ITS2 regions of ribosomal RNA of *Beauveria bassiana* (AB576868, MG548313, and LC768985) with ITS sequences generated from *B. bassiana* stock strains GHA, NI8, JG1, SPE and fungal cultures recovered from cotton squares tissue from plants inoculated with stock fungal isolates. Nucleotide polymorphisms, insertions, and deletions in the nucleotide sequences are shown in white text with black background.

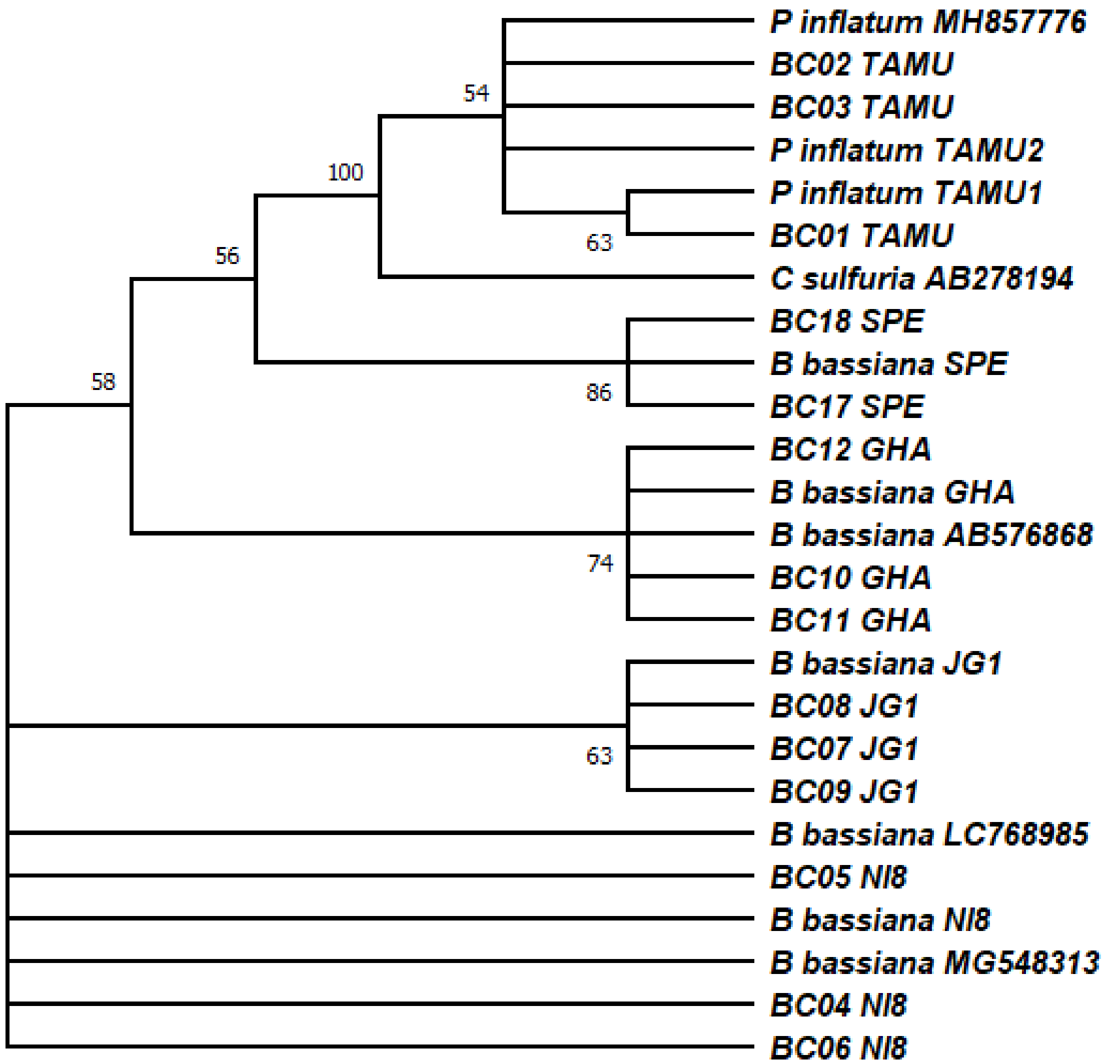

Figure S2. The evolutionary history was inferred among internal transcribed spacer 1 (ITS1), 5.8S and ITS2 regions using Maximum Likelihood based on the Kimura 2-parameter model of sequence evolution [59]. The database sequences of ITS regions of *Beauveria bassiana* (AB576868, MG548313, and LC768985) and *Phialemonium inflatum* (MH857776) were used as reference to generate evolutionary relationships between stock strains GHA, NI8, JG1, SPE, and TAMU and fungal cultures recovered from cotton squares obtained from plants inoculated with stock fungal isolates. *Cephalotheca sulfurea* ITS sequence (AB278194) was used as the outgroup. GenBank accessions and strain names are provided at branch tips. Tree was based on approximatelya 696 nucleotide-long consensus alignment. The bootstrap consensus at each node was inferred from 10,000 replicates [60] and partitions with less than 50% bootstrap support are collapsed. Neighbor-Join and BioNJ algorithms were applied to a matrix of pairwise distances estimated using the Maximum Composite Likelihood (MCL) approach and the topology with superior log likelihood value was selected to obtain the initial tree(s). A discrete Gamma distribution was used to model evolutionary rate differences among sites (5 categories (+*G*, parameter = 0.3573). Evolutionary analyses were conducted in MEGA11 [56].
